# Identification of biomarkers associated with programmed cell death in liver ischemia–reperfusion injury: insights from machine learning frameworks and molecular docking in multiple cohorts

**DOI:** 10.3389/fmed.2025.1501467

**Published:** 2025-03-14

**Authors:** Jifeng Liu, Yeheng Jin, Fengchen Lv, Yao Yang, Junchen Li, Yunshu Zhang, Lei Zhong, Wei Liu

**Affiliations:** ^1^Department of General Surgery, First Affiliated Hospital of Dalian Medical University, Dalian, Liaoning, China; ^2^Department of Second Clinical College, China Medical University, Shenyang, Liaoning, China; ^3^Department of Traditional Chinese Medicine, First Affiliated Hospital of Dalian Medical University, Dalian, Liaoning, China

**Keywords:** liver ischemia–reperfusion injury, programmed cell death, molecular docking, machine learning, biomarkers

## Abstract

**Introduction:**

Liver ischemia-reperfusion injury (LIRI) is a major reason for liver injury that occurs during surgical procedures such as hepatectomy and liver transplantation and is a major cause of graft dysfunction after transplantation. Programmed cell death (PCD) has been found to correlate with the degree of LIRI injury and plays an important role in the treatment of LIRI. We aim to comprehensively explore the expression patterns and mechanism of action of PCD-related genes in LIRI and to find novel molecular targets for early prevention and treatment of LIRI.

**Methods:**

We first compared the expression profiles, immune profiles, and biological function profiles of LIRI and control samples. Then, the potential mechanisms of PCD-related differentially expressed genes in LIRI were explored by functional enrichment analysis. The hub genes for LIRI were further screened by applying multiple machine learning methods and Cytoscape. GSEA, GSVA, immune correlation analysis, transcription factor prediction, ceRNA network analysis, and single-cell analysis further revealed the mechanisms and regulatory network of the hub gene in LIRI. Finally, potential therapeutic agents for LIRI were explored based on the CMap database and molecular docking technology.

**Results:**

Forty-seven differentially expressed genes associated with PCD were identified in LIRI, and functional enrichment analysis showed that they were involved in the regulation of the TNF signaling pathway as well as the regulation of hydrolase activity. By utilizing machine learning methods, 11 model genes were identified. ROC curves and confusion matrix from the six cohorts illustrate the superior diagnostic value of our model. MYC was identified as a hub PCD-related target in LIRI by Cytoscape. Finally, BMS-536924 and PF-431396 were identified as potential therapeutic agents for LIRI.

**Conclusion:**

This study comprehensively characterizes PCD in LIRI and identifies one core molecule, providing a new strategy for early prevention and treatment of LIRI.

## 1 Introduction

Liver ischemia–reperfusion injury (LIRI) is a major reason for liver injury in surgical procedures such as hepatectomy and liver transplantation (LT) (1). LIRI involves two phases: localized ischemic injury and reperfusion injury, which are interrelated (2). In addition, LIRI can be categorized into warm LIRI and cold LIRI. Warm LIRI occurs in cases of transplantation, trauma, shock, and elective liver surgery, where the liver blood supply is temporarily interrupted. Cold LIRI occurs during the organ preservation period prior to transplantation. Several factors contribute to LIRI, including activation of Kupffer cells, oxidative stress, and upregulation of pro-inflammatory cytokine signaling (3). LIRI involves a wide range of cellular and biochemical processes, including dysregulation of the normal phenotype of every liver cellular component (1). However, a large proportion of these processes remain unknown or unclear. LIRI is an unavoidable process in LT, which may not only lead to early graft dysfunction and chronic rejection but also increase the risk of liver tumor recurrence and fibrosis (4–6). Despite considerable efforts in therapeutic strategies for LIRI, prevention is the primary strategy for reducing LIRI (7). Therefore, it is imperative to investigate novel molecular mechanisms and key targets for LIRI.

Programmed cell death (PCD), also known as regulated cell death, is a specified form of cell death that could be controlled by different biomolecules (8). PCD is crucial for preserving tissue homeostasis and getting rid of unhealthy or superfluous cells. It has become a crucial disorder phenotype and has received a lot of attention in many disorders (9–11). Numerous studies have indicated that cell death is the main potential mechanism of LIRI (1, 12). In the past decades, it was believed that necrosis and apoptosis occurred mainly in LIRI (13). Numerous novel forms of PCD, like ferroptosis, cuproptosis, disulfidptosis, and pyroptosis, have been identified in recent years. LIRI can initiate a variety of cellular processes that are related to multiple types of PCD (14). For example, it was found that the iron death marker Ptgs2 was significantly upregulated in the mouse LIRI model (15). Meanwhile, chronic exposure to excess Cu leads to an increase in ROS and promotes apoptosis in mouse liver (16). It was also found that pyroptosis and NLRP3 targeting can reduce LIRI (17). In addition, PCD is strongly correlated with the severity of LIRI injury, which also implies that PCD has an important role in the treatment of LIRI (14). However, how to effectively inhibit PCD and reduce the cascade of cell death in LIRI remains a challenge, and a deeper understanding of these cell death patterns is needed to identify more precise targets and therapeutic agents.

Recently, the emergence of bioinformatics, machine learning, and molecular docking technologies has accelerated the processing and analysis of large-scale data to efficiently identify hub molecules and potential therapeutic agents of diseases (18, 19). The study will utilize bioinformatics, machine learning and molecular docking techniques to comprehensively explore the expression and mechanism of PCD-related genes in LIRI and provide a theoretical basis for future studies of PCD in LIRI.

## 2 Methods

### 2.1 Data collection

The datasets analyzed in this study were downloaded from the GEO database. The GSE12720 (20) contains 21 preoperative samples and 21 post reperfusion samples, GSE112713 (21) contains 11 preoperative samples and 11 post reperfusion samples, and GSE14951 (22) contains 5 preoperative samples and 5 post reperfusion samples. They were merged as a training set by the “ComBat” function from “sva” package (23). Principal component analysis (PCA) was performed to verify the batch effect before and after the data operation. PCA is a method of data dimensionality reduction, which extracts the feature vectors (components) of data from high latitude data, converts them into low dimensional data, and displays these features using two-dimensional or three-dimensional graphs. The GSE23649 (33 LIRI and 33 control samples), GSE15480 (6 LIRI and 6 control samples), and GSE151648 (24) (40 LIRI and 40 control samples) datasets were used as validation sets for subsequent biomarker validation.

The PCD-related genes were carefully hand-curated from a variety of reliable sources, such as the KEGG database, the MSigDB database, review articles, and manual curation (8, 25–29). A total of 1,567 genes associated with 19 types of PCD were eventually included, as shown in Supplementary Table S1.

### 2.2 Analysis of differences between LIRI and normal samples

Using the “limma” software, the criteria for detecting differentially expressed genes (DEGs) between LIRI and normal samples were set to |log2 fold change (FC)| > 0.585 and adjust *p* < 0.05 (18). The expression differences for LIRI were visualized by a volcano plot using the “ggplot2” package in R and a heatmap using the “pheatmap” package in R. The proportions of various immune cell types were evaluated using ssGSEA from the “GSVA” package in order to compare the differences in immunity status between groups (30). Gene Set Enrichment Analysis (GSEA) is a computational method used to determine differences in biological process activity or pathway enrichment across different samples. It evaluates whether predefined sets of genes are statistically significantly associated with a biological state. In this study, all genes were first categorized into two groups based on their positive and negative logFC values. We then performed enrichment analysis on the positive and negative logFC gene groups using the “clusterProfiler” package in R (31).

### 2.3 Functional enrichment analysis of PCD-related DEGs

The Venn map was applied to select the common genes between DEGs and PCD-related genes. The STRING database^1^ was used to investigate protein interactions, with the validity of such interactions being determined by a composite score greater than 0.4. The GeneMANIA database^2^ prioritized genes for functional tests. Finally, the Metascape database^3^ was used to conduct functional enrichment analysis (32).

### 2.4 Using machine learning to screen characteristic genes

Different machine learning methods have different characteristics in data processing and pattern recognition, and their combined use can more comprehensively mine the potential information in the data, while avoiding the bias that may be brought by relying on a single method. We used 12 machine learning methods (Lasso, Ridge, Stepglm, XGBoost, Random Forest, Enet, plsRglm, GBM, Naïve Bayes, LDA, glmBoost, and SVM) to build the diagnostic model. 113 combinations of these 12 techniques were investigated through methodical designs for model generation and variable selection utilizing the training dataset (33). Three datasets were then used as external test datasets to validate the model’s performance. We also calculated the AUC values of each model and output the AUC matrix, and then used “ComplexHeatmap” to draw the AUC heatmap for visualizing the performance of each model on the training and testing sets. The best model is the one with the highest average AUC over training and testing cohorts (29).

### 2.5 Potential functions of the hub gene in LIRI

To identify the hub gene, the cytoHubba plug in Cytoscape was applied (34). GSEA and GSVA were utilized to elucidate the molecular mechanisms between high- and low-hub gene expression samples (35). Meanwhile, the ssGSEA was also used to assess the correlation between significantly different enriched immune cell types and the hub gene. These gene sets used in these analyses were obtained from MSigDB.

### 2.6 Construction of the transcription factor (TF) and ceRNA networks of hub gene

We utilize TFTF online (36) to predict the hub gene for TFs based on the integration of five major TF-Target online tools, namely hTFtarget (37), ENCODE (38), JASPAR (39), GTRD (40), and ChIP_Atlas (41). In addition, the TargetScan, miRDB, and miRanda databases were used to anticipate miRNA-mRNA pairs in order to identify the ceRNA network that might be influenced by model genes (42). Genes that were simultaneously listed in three databases were the only ones that were thought to be possible mRNA targets for further research (43). To predict miRNA-lncRNA pairs, the spongeScan database was used. At last, the ceRNA network could be seen using Cytoscape (44). Meanwhile, the Human Protein Atlas (HPA^4^) was utilized to examine the hub gene’ immunofluorescence and single-cell type atlases.

### 2.7 Identifying potential small-molecule compounds for the treatment of LIRI

The CMap database^5^ can link diseases, genes, and drugs based on gene expression profiles. The upregulated DEGs of LIRI were entered into the CMap database to identify potential small-molecule compounds for the treatment of LIRI (43). Then, the protein structures of the feature genes were obtained from the PDB database, and the AutoDock tool was utilized to compute the hydrogenation and charge of proteins. PubChemdatabase to download the chemical structure of the drug’s active ingredient. The AutoDock is used to examine the rotatable bonds and charge balance of small molecules. Finally, PyMol software was used to check the docking complex.

## 3 Results

### 3.1 Gene expression and biological characteristics of patients with LIRI

**Figure 1:**
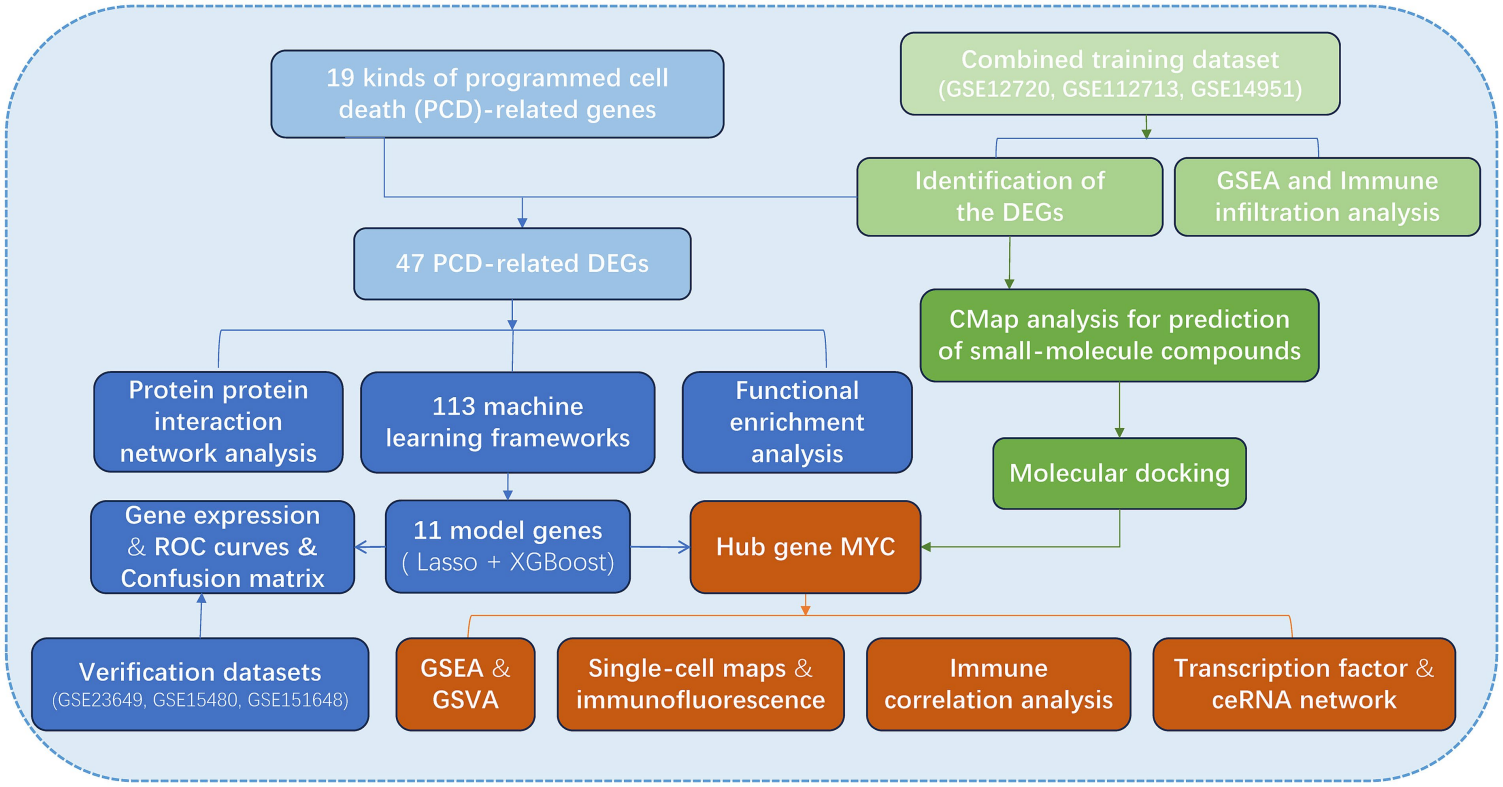
The process of data analyzing in this study.

**Figure 2:**
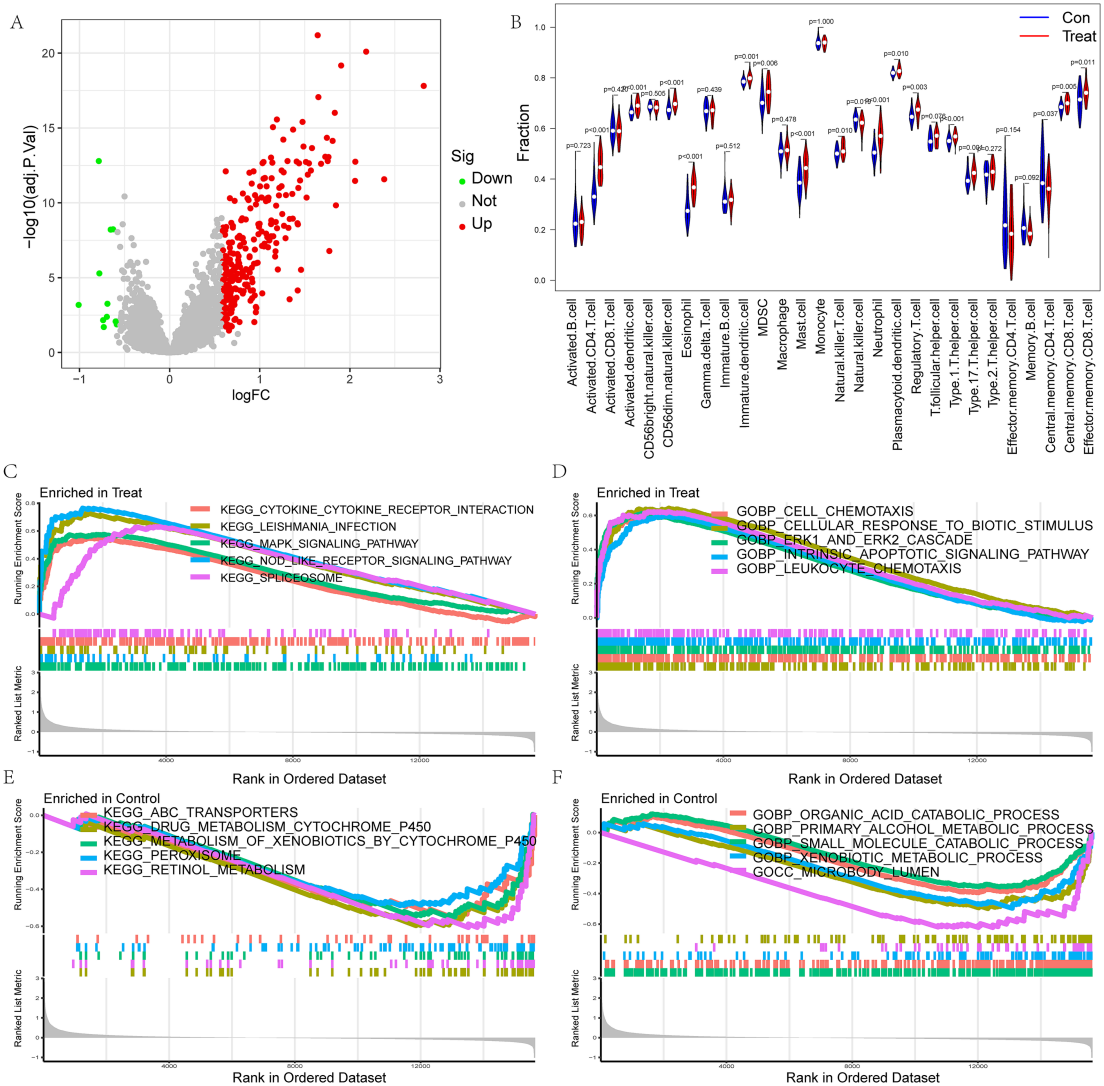
Analysis of differences between LIRI and control samples. **(A)** The volcano plot for DEGs in LIRI; **(B)** Differences in immune cells between LIRI and controls; **(C–F)** GSEA analysis of KEGG and GO between LIRI and controls.

The flow chart of the research is presented in Figure 1. Initially, we merged three datasets of LIRI and corrected batch effects using the “sva” software package. As can be seen from Supplementary Figures S1A–D, after data normalization, the difference between batches is effectively eliminated, and the three datasets can be merged. The volcano map and heatmap showed the DEGs for the LIRI (Figure 2A and Supplementary Figure S1E). Given the important role of immune cells in LIRI, differences in immune cell infiltration were subsequently compared between LIRI patients and controls. It was found that LIRI patients had a higher percentage of activated CD4 T cells, dendritic cells, eosinophils, MDSC, mast cells, NK T cells, neutrophils, T helper cells, and CD8 T cells, while central memory CD4 T cells and NK cells were in contrast (Figure 2B). Further GSEA analysis was then performed to explore the differences in biological function between LIRI patients and controls. The results showed that relevant pathways and functions such as apoptotic and cytokines were significantly upregulated in the LIRI group (Figures 2C,D), while metabolism-related pathways and functions were significantly upregulated in the control group (Figures 2E,F).

### 3.2 PPI network and functional enrichment analysis for PCD-related DEGs

**Figure 3:**
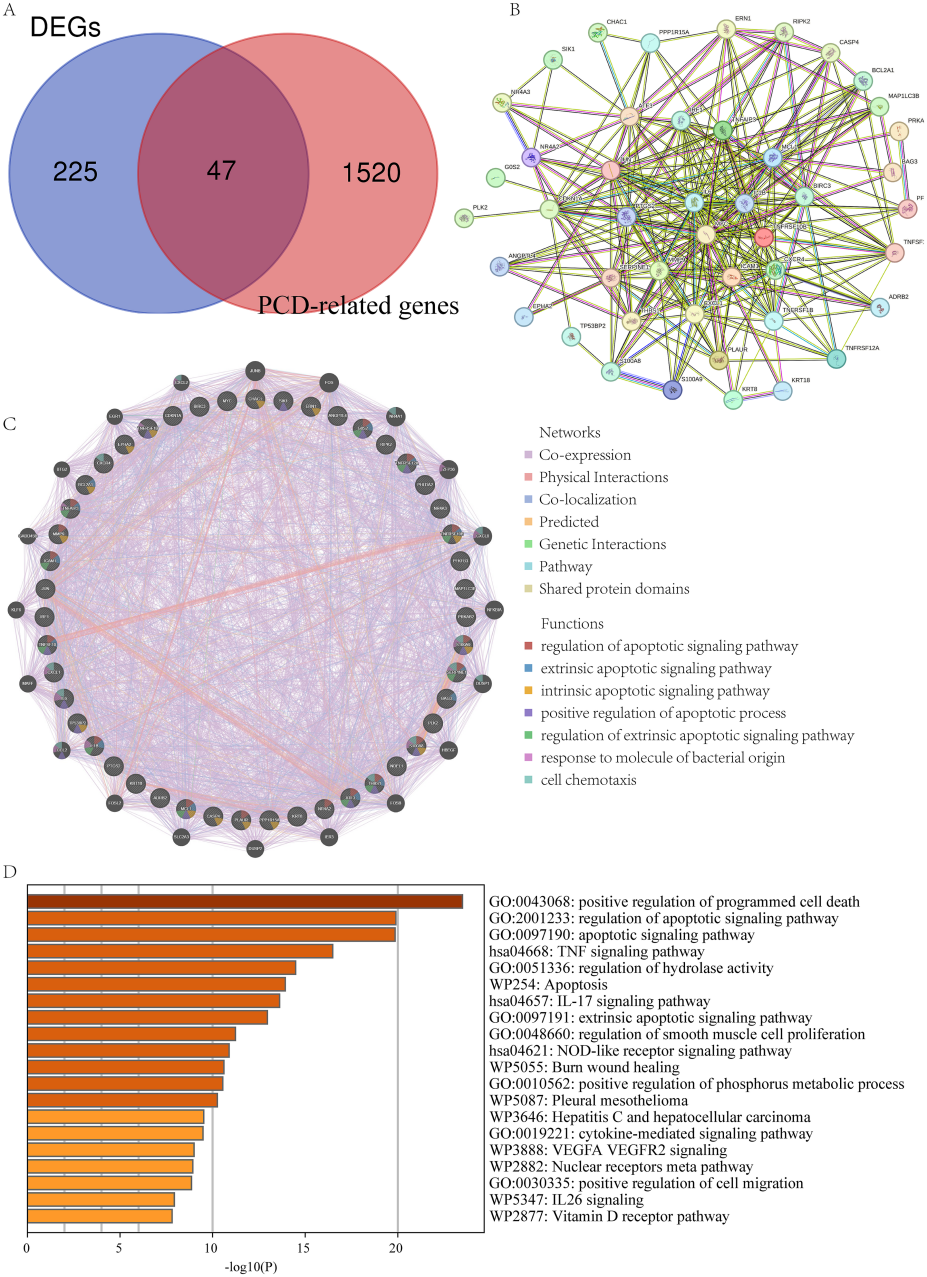
Functional enrichment analysis of PCD-related DEGs. **(A)** The intersection of DEGs and PCD-related gene; **(B)** PPI of the PCD-related DEGs; **(C)** GeneMANIA analysis of PCD-related DEGs; **(D)** Functional enrichment analyses by the Metascape database.

To further explore the role of PCD in LIRI, we collected PCD-related genes from 19 types of PCD. Using the Venn diagram, the DEGs and PCD-related genes were crossed, and 47 PCD-related DEGs were obtained (Figure 3A). The PPI network complex was constructed by importing 47 PCD-related DEGs into the STRING database. PPI analysis shows tight junctions between these genes (Figure 3B). Then, we used GeneMANIA to predict correlations between colocalization, pathways, shared protein domains, co-expression, and prediction. The network showed that these genes were mainly enriched in the regulation of apoptotic signaling pathway, response to molecules of bacterial origin, and cell chemotaxis (Figure 3C). Finally, the Metascape database was used to explore the biological activities and pathways of PCD-related DEGs. The result showed that they were involved in the regulation of PCD, the TNF signaling pathway, and the regulation of hydrolase activity (Figure 3D).

### 3.3 Selection of characteristic genes via machine learning algorithms

**Figure 4:**
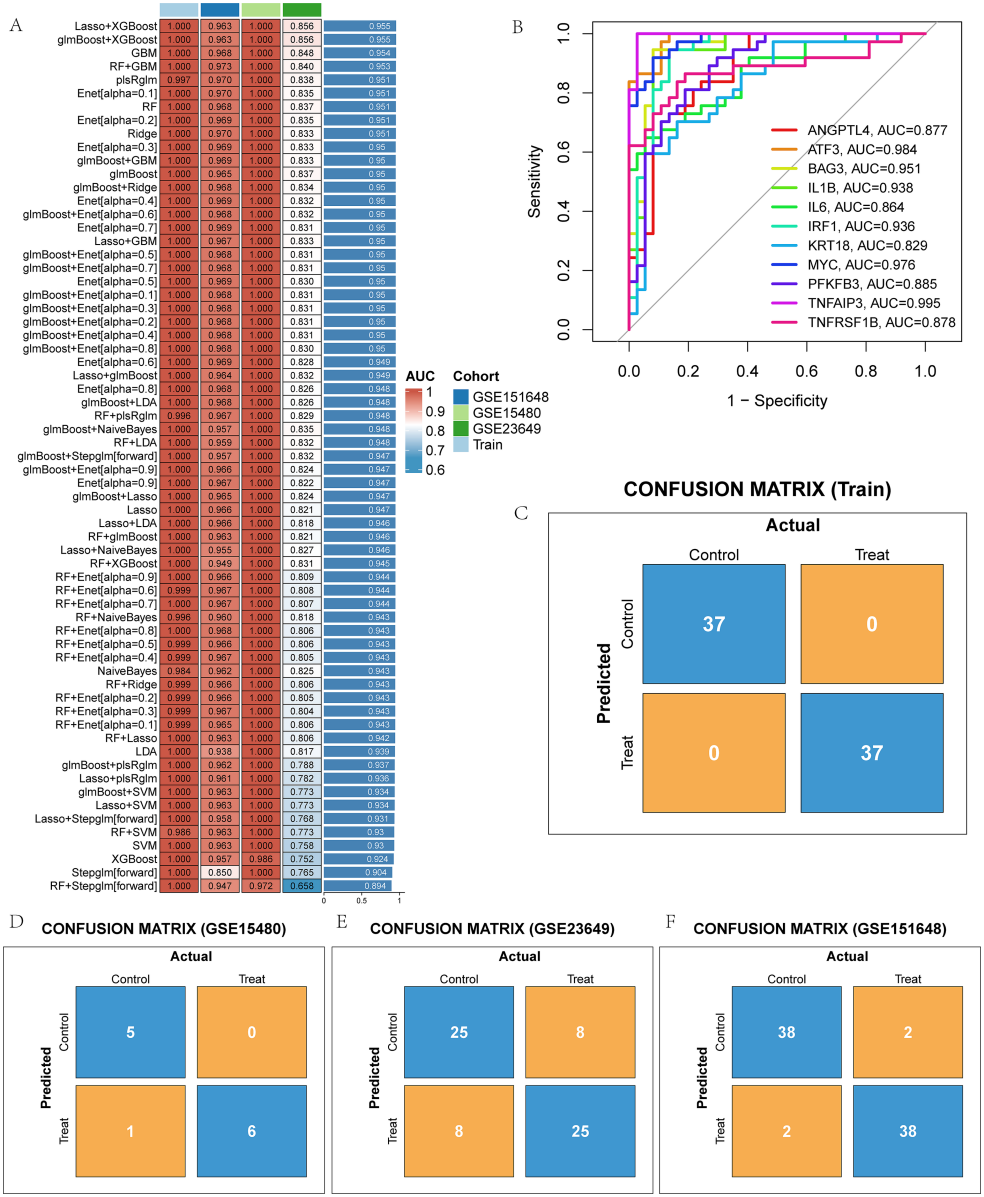
The model genes of LIRI were screened by machine learning method. **(A)** AUC values of 113 machine learning models; **(B)** ROC curves of diagnostic performance of 11 model genes for LIRI; **(C–F)** Confusion matrix of the model in training and three test sets.

A total of 12 machine learning algorithms were combined to identify the most robust diagnostic model based on 47 PCD-related DEGs. Among them, the Lasso + XGBoost machine learning method has the highest average AUC value in the training set and the three validation sets, so the Lasso + XGBoost model is chosen as the final prediction model (Figure 4A and Supplementary Figures S2A–D). We identified 11 model genes: ANGPTL4, ATF3, BAG3, IL1B, IL6, IRF1, KRT18, MYC, PFKFB3, TNFAIP3, and TNFRSF1B. ROC curves of 11 model genes were shown in Figure 4B. The confusion matrix shows that our diagnostic model has high sensitivity, specificity and accuracy (Figure 4C), and it was confirmed in the verification sets (Figures 4D–F).

### 3.4 Selection of the hub gene by the Cytoscape

**Figure 5:**
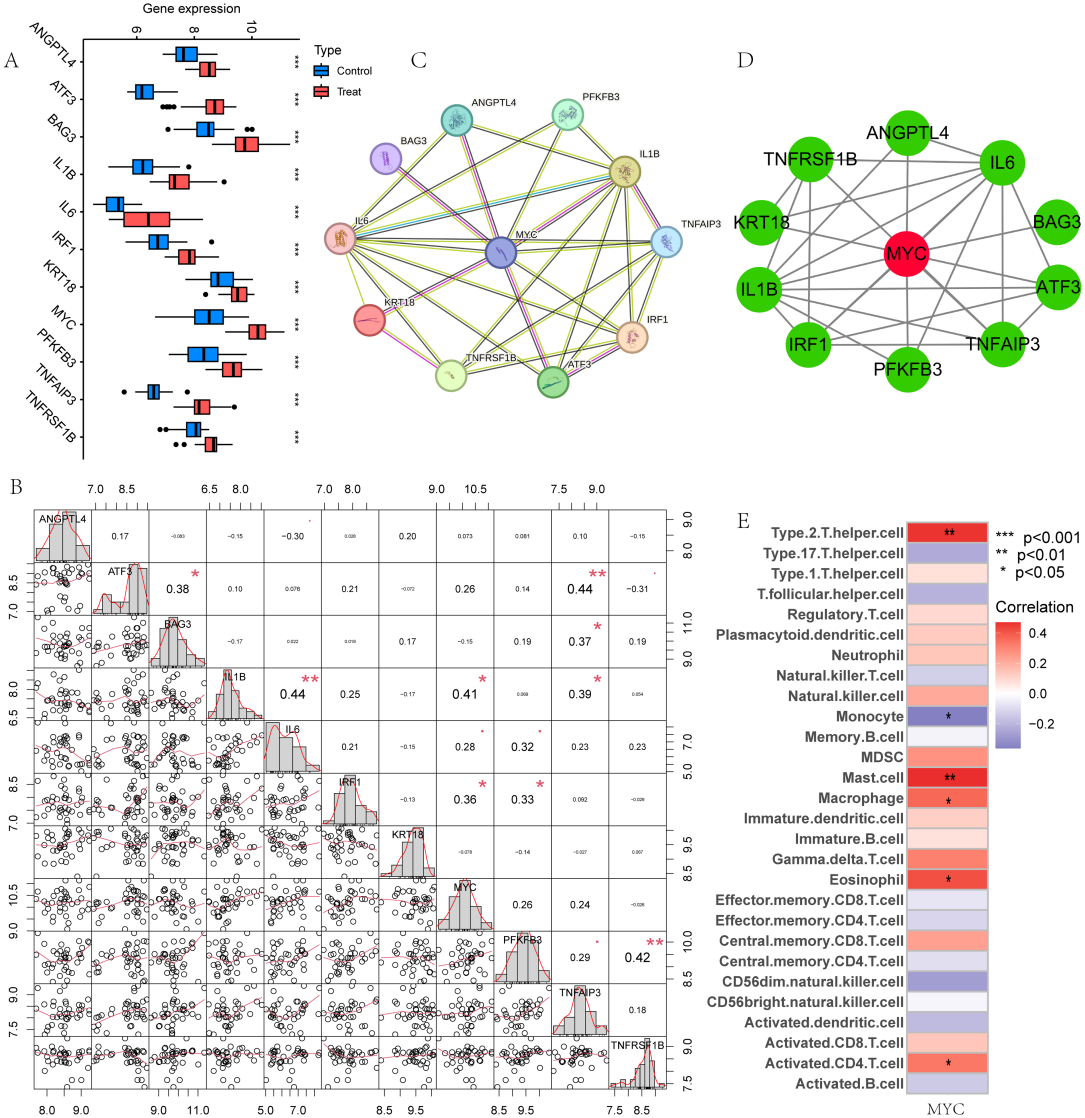
Identification of the hub gene. **(A)** Box plot showed the expression difference of model genes between LIRI and control samples in training set. **(B)** Correlation analysis of 11 model genes; **(C)** PPI of the model genes; **(D)** The hub gene MYC was identified from 11 genes using Cytoscape; **(E)** Immune cell correlation analysis of MYC.

We then performed gene expression analysis for these 11 model genes in the training set and validation sets, respectively. Analysis in the training set showed that these 11 model genes were all highly expressed in LIRI patients (Figure 5A). The gene expression analysis of the validation set was shown in Supplementary Figures S3A–C, which was consistent with the training set. Figure 5B demonstrated the correlation between these 11 model genes. To further explore the most critical of these genes, we examined the PPI networks of model genes using the STRING tool (Figure 5C) and inserted the results into Cytoscape. MYC was detected as the hub gene for these model genes using the Cytohubba plugin (Figure 5D).

### 3.5 Immunological characteristics and biological function of the MYC in LIRI samples

**Figure 6:**
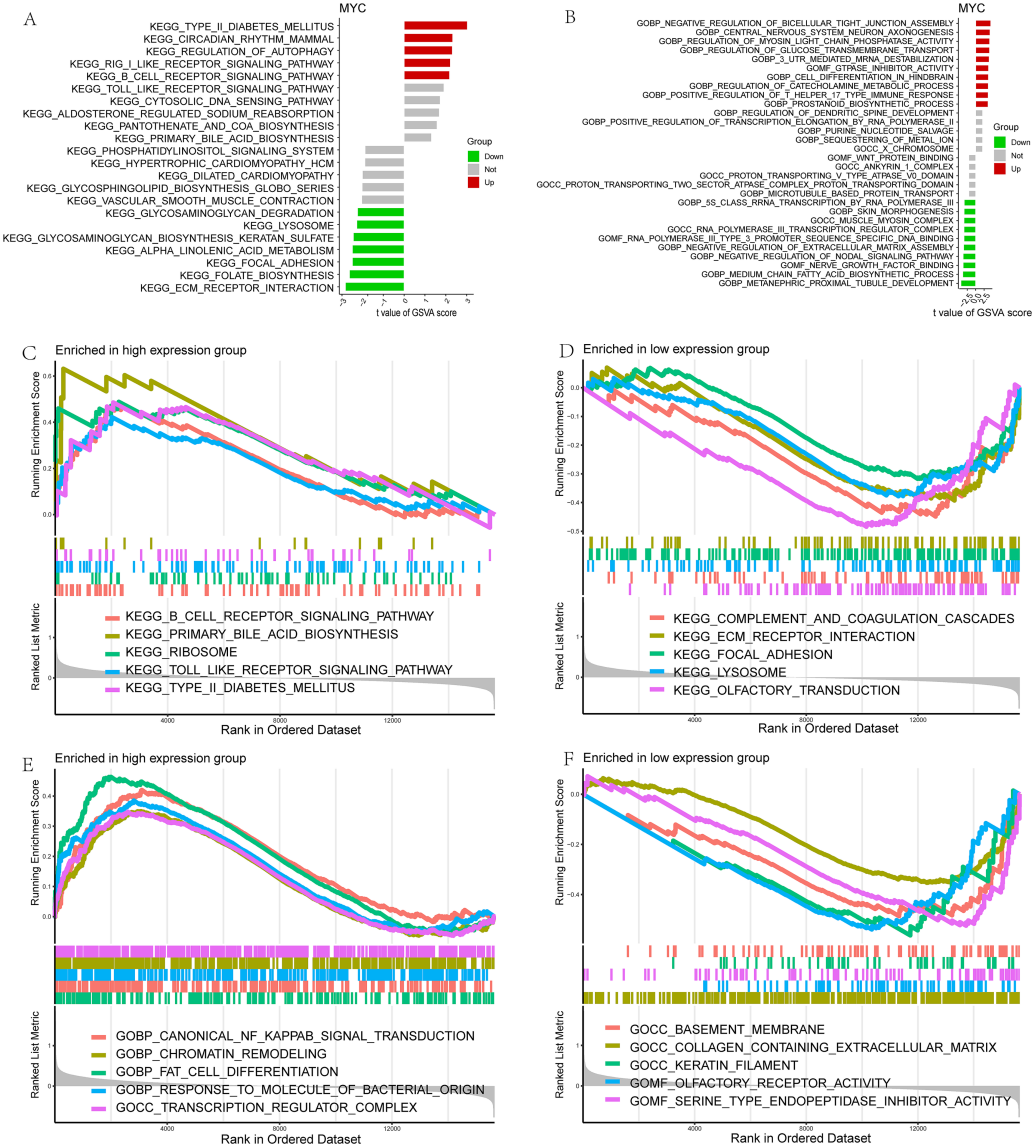
Functional enrichment analysis of MYC in LIRI. **(A,B)** Enrichment biological functions and pathways of MYC identified by GSVA; **(C–F)** Enrichment biological functions and pathways of MYC identified by GSEA.

Next, the relationship between MYC and the different immune cells was further studied. It showed that MYC was significantly positively correlated with Type 2 T helper cells, mast cells, macrophages, eosinophils and activated CD4 T cells, while oppositely in monocytes (Figure 5E). Then we performed GSVA analysis to further explore the biological functions of hub genes in LIRI. MYC was significantly positively associated with circadian rhythm, regulation of autophagy, and regulation of immune response-related pathways and functions in LIRI samples (Figures 6A,B). Subsequently, GSEA analysis was utilized to further explore the possible mechanism of action of MYC in LIRI samples. It showed that MYC was significantly positively associated with B cell receptor, Toll like receptor, and transcription regulator complex-related pathways and functions, while oppositely in ECM receptor interaction, complement and coagulation cascades, and basement membrane-related pathways and functions (Figures 6C–F).

### 3.6 The transcription factors (TFs), ceRNA networks, single-cell maps, and immunofluorescence analysis of MYC

**Figure 7:**
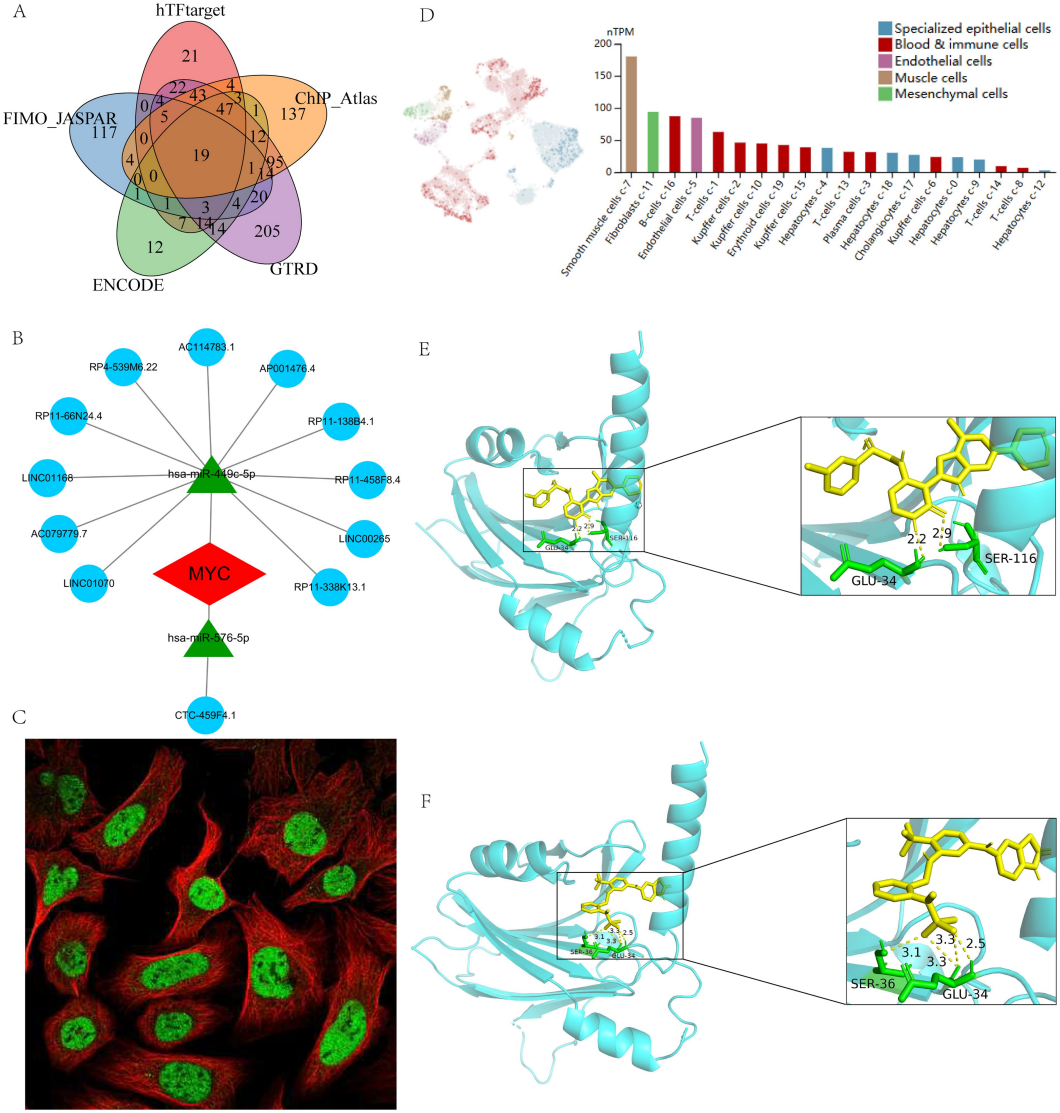
Regulatory network, single-cell mapping and immunofluorescence analysis of MYC. **(A)** Prediction of TFs based on different databases; **(B)** The ceRNA network of MYC; **(C)** The immunofluorescence of MYC based on HPA database; **(D)** The single-cell mapping of MYC based on HPA database; **(E)** Molecular docking of BMS-536924 and MYC; **(F)** Molecular docking of PF-431396 and MYC.

TFs are critical in regulating gene expression and shaping cellular and organismal phenotypes. We therefore combined five databases to characterize TFs in MYC and identified a total of 19 reliable TFs: CTCF, BATF, EGR1, MAZ, PAX5, SP1, TCF12, E2F6, FOS, JUND, TEAD4, NFIC, PBX3, STAT3, TFAP2A, TFAP2C, TCF7L2, FOSL2, and RELA (Figure 7A). In addition, the ceRNA network of MYC was built using various public databases. Ultimately, we were able to identify 2 objective miRNAs and 12 objective lncRNAs of MYC (Figure 7B). The network disclosed transcriptional regulatory mechanisms for MYC. The cellular location of MYC was then investigated. It can be found that MYC is mainly expressed in the nucleoplasm, where green represents the target protein and red represents microtubules (Figure 7C). In addition, we evaluated MYC expression at the single-cell data level using the HPA database. It was found that MYC was mainly distributed in smooth muscle cells and fibroblasts (Figure 7D).

### 3.7 Identification of potential small-molecule compounds for the treatment of LIRI

In addition, to search for potential drugs to treat LIRI, we entered upregulated DEGs in LIRI into the CMap database for analysis. The top 10 small-molecule compounds are shown in Table 1 and are considered potential therapeutic drugs for LIRI. Then we conducted molecular docking analysis of these 10 small-molecule compounds with MYC. The minimum binding energy between the hub gene and each small-molecule compound is shown in Table 1. The results showed that the minimum binding energy between the ligand and the receptor was less than −7.0 kcal/mol, so the target proteins had a good affinity for the active ingredient, suggesting that these small-molecule drugs could act on MYC to treat LIRI. Among them, BMS-536924 (−9.8 kcal/mol) and PF-431396 (−9.4 kcal/mol) have the lowest free binding energy for MYC and are most likely to be potential therapeutic agents for LIRI (Figures 7E,F).

**Table 1:** Potential treatment options for LIRI analyzed by CMap and molecular docking.

pert_iname	moa	target_name	raw_cs	fdr_q_nlog10	norm_cs	Free binding energy (kcal/mol)
Alvocidib	CDK inhibitor	CDK2|CDK4|CDK1|CDK6|CDK7|CDK9|CDK5|CDK8|EGFR|PYGM|BCL2|BIRC5|CCNT1|MCL1|XIAP	−0.79	15.6536	−2.9083	−8.7
TWS-119	GSK inhibitor	GSK3B|JUN|MYC	−0.707	15.6536	−2.603	−9.1
Lapatinib	EGFR inhibitor|ERBB2 inhibitor	EGFR|ERBB2|CYP3A5	−0.6816	15.6536	−2.5093	−9.1
PF-431396	Focal adhesion kinase inhibitor	PTK2|PTK2B	−0.6809	15.6536	−2.5069	−9.4
BMS-536924	IGF-1 inhibitor	IGF1R|INSR|AKT1|CCNE1|CDK2|CYP3A4|ERBB2|KDR|LCK|MAPK1|MET|PDGFRA|PDGFRB	−0.6756	15.6536	−2.4874	−9.8
U-0126	MEK inhibitor	MAP2K1|MAP2K2|JAK2|AKT1|CHEK1|GSK3B|LCK|MAP2K7|MAPK1|MAPK11|MAPK12|MAPK14|MAPK8|PRKCA|RAF1|ROCK1|RPS6KB1|SGK1	−0.6751	15.6536	−2.4854	−7.6
Selumetinib	MEK inhibitor	MAP2K1|MAP2K2	−0.674	15.6536	−2.4814	−8.2
ER-27319	Syk inhibitor	SYK	−0.6708	15.6536	−2.4695	−8.0
WZ-3146	EGFR inhibitor	EGFR	−0.6683	15.6536	−2.4603	−9.1
A-443654	AKT inhibitor	AKT1|AKT2	−0.6499	15.6536	−2.3925	−8.8

## 4 Discussion

LIRI is one of the serious complications and a major risk factor for early allograft failure after LT, which has led to a shortage of transplanted organs (45). LIRI is a self-amplifying process involving two interrelated phases of ischemic and reperfusion injury (46). The ischemic insult is characterized by a dysfunction of the mitochondrial respiratory chain, activation of cell death programs, and alterations in expression of genes (47, 48). The field of LT has prioritized research to develop effective strategies to minimize IRI because LIRI is linked to increased short- and long-term morbidity and death (49). PCD is a specified form of cell death that could be controlled by different biomolecules. Numerous studies have demonstrated that PCD is closely correlated with the severity of LIRI injury, which also implies that PCD has an important role in the treatment of LIRI (14). The aim of this study was to conduct a preliminary exploration of PCD patterns in LIRI using integrated bioinformatics analysis and molecular docking techniques to identify central genes and potential therapeutic agents to enhance early prevention and treatment of LIRI.

In the current study, we comprehensively searched for LIRI-related datasets and eventually included a total of six cohorts of LIRI samples. To understand the underlying mechanisms of LIRI occurrence, we first compared the differences in biological function between LIRI and controls by GSEA analysis. The results showed that the intrinsic apoptotic signaling pathway was significantly upregulated in the samples of the LIRI group. Given that different forms of PCD are widely cross-talked and play important roles in LIRI, we collected a total of 1,567 genes for 19 types of PCD to further comprehensively explore the potential mechanisms and therapeutic targets of PCD in LIRI. Among them, 47 PCD-related genes were differentially expressed in LIRI, and these PCD-related DEGs were used to follow up further studies. In order to explore the potential mechanism of these genes in LIRI, we performed functional enrichment analyses of 47 PCD-related DEGs. The result showed that the DEGs were involved in the positive regulation of PCD, apoptotic signaling pathway, and TNF signaling pathway. These genes may contribute to the development of LIRI by regulating PCD, TNF signaling pathways, and other pathways.

Then, we applied 113 machine learning combinations to screen out 11 characteristic genes as diagnostic model. We used multiple analytical methods to further evaluate the value of our diagnostic model in LIRI, which showed specific expression and a high degree of prediction power. The average AUC value of the model was 0.955 in the training set and in three different external validation sets. Although this may be related to the small sample size, and the validation of multiple cohorts is sufficient to illustrate the excellent performance of the model, suggesting that these 11 genes can be used as diagnostic biomarkers for LIRI patients.

Subsequently, Cytoscape was utilized to further screen these 11 genes for the hub gene MYC, which is considered a hub PCD-related target in LIRI. MYC is a pleiotropic transcription factor involved in multiple cellular processes. MYC is thought to regulate more than 15% of human genes and is sometimes described as a “master gene regulator.” It is involved in the regulation of a variety of cellular processes, including cell growth, cell cycle, differentiation, apoptosis, angiogenesis, DNA repair, and stem cell formation (50). Recent studies have shown that MYC promotes renal tubular cell apoptosis in ischemia–reperfusion-induced kidney injury by regulating the apoptotic pathway (51). In addition, Xiao et al. (52) found that inhibition of the MYC/PTEN axis attenuates cerebral ischemia–reperfusion injury in the rat. Sun et al. (53) found that MYC may regulate NDRG2 expression in cardiomyocytes and promote apoptosis in rat cardiomyocytes after ischemia–reperfusion. These studies suggest that MYC may play an important role in ischemia–reperfusion injury; however, the role of MYC in LIRI has not been reported.

To further explore the potential mechanisms of MYC action in LIRI, we conducted a comprehensive analysis. MYC was found to be significantly positively correlated with Type 2 T helper cells, mast cells, macrophages, eosinophils and activated CD4 T cells. Enrichment analysis showed that B cell receptor, Toll like receptor, and transcription regulator complex-related pathways and functions were significantly upregulated in high-MYC patients. Previous studies have shown that MYC expression in immune cells is tightly regulated during development and in response to immune stimulation (54). MYC plays a crucial role in governing the development, differentiation, and activation of immune cells. By driving the expression of a broad range of metabolic genes, MYC orchestrates metabolic programs that support immune function. For example, research has demonstrated that MYC is involved in regulating T cell development, activation, and differentiation, thereby facilitating adaptive immune responses (55). Our results suggested that MYC may contribute to LIRI by regulating these immune cells and pathways. Subsequent TF prediction and construction of the ceRNA network further revealed the potential regulatory network of MYC, providing a scientific basis for further studies of MYC in LIRI in the future.

In addition, MYC expression was significantly up-regulated in LIRI, so it may be new therapeutic targets for LIRI. We molecularly docked the top ten predicted small-molecule compounds with MYC, respectively, and found that BMS-536924 and PF-431396 had the strongest binding ability to the MYC, suggesting that BMS-536924 and PF-431396 could be used to treat LIRI by binding to MYC. BMS-536924 is an effective IGF-I small molecule inhibitor, while PF-431396 is a FAK small molecule inhibitor. Previous research has demonstrated that IGF-II knockdown can suppress MYC expression in hepatocellular carcinoma cells via the FAK/PI3K/AKT signaling pathway (56). In line with this, Zhang et al. (57) found that FAK inhibitors can inhibit MYC expression by disrupting the PI3K/AKT axis. Additionally, studies have shown that IGF-1 overexpression can enhance MYC expression (58), and MYC also can promote tumor cell growth through paracrine signaling mediated by the IGF-1/IGF-1R axis (59). These studies provide strong theoretical support for the possibility that both BMS-536924 (IGF-1 inhibitor) and PF-431396 (FAK inhibitor) could treat LIRI by modulating MYC, suggesting that targeting MYC with these small molecules may offer a promising therapeutic strategy for LIRI.

This is the first bioinformatics study to uncover the potential role of PCD in LIRI. However, our study has some limitations. First, although we included multiple datasets for analysis and validation, clinical studies are needed in the future to support our conclusions. In addition, further *in vivo* and *in vitro* experiments are needed to verify the mechanisms of action of MYC and the therapeutic effects of BMS-536924 and PF-431396 in LIRI in the future.

## 5 Conclusion

In conclusion, by integrating a series of bioinformatics approaches, we identified MYC as reliable diagnostic and therapeutic targets for LIRI and comprehensively analyzed its mechanisms of action in the LIRI. More importantly, we found BMS-536924 and PF-431396 may be potential drugs for the treatment of LIRI by acting on MYC.

## Data Availability

The datasets analyzed in this work can be found in the GEO database https://www.ncbi.nlm.nih.gov/geo/ (GSE12720, GSE112713, GSE14951, GSE23649, GSE15480, GSE151648). Further inquiries can be directed to the corresponding author.

## Footnotes

^1^ https://cn.string-db.org/

^2^ https://genemania.org/

^3^ https://metascape.org/

^4^ https://www.proteinatlas.org/

^5^ https://clue.io/

## Supplementary material

Supplementary Figure S1 **(A,B)** Box plot of expression level before and after merging of datasets; **(C,D)** PCA analysis before and after merging of datasets; **(E)** The heat map for DEGs in LIRI.

Supplementary Figure S2 **(A–D)** The ROC curves of our model in training and three test sets; **(E–G)** Box plots showed the expression difference of model genes between LIRI and control samples in test sets.

Supplementary Table S1 List of 19 PCD-related genes.

## References

[1] Peralta C Jimenez-Castro MB Gracia-Sancho J. Hepatic ischemia and reperfusion injury: effects on the liver sinusoidal milieu. J Hepatol. (2013) 59:1094–106. doi: 10.1016/j.jhep.2013.06.017, PMID: 23811302

[2] Hirao H Nakamura K Kupiec-Weglinski JW. Liver ischaemia-reperfusion injury: a new understanding of the role of innate immunity. Nat Rev Gastroenterol Hepatol. (2022) 19:239–56. doi: 10.1038/s41575-021-00549-8 34837066

[3] Papadopoulos D Siempis T Theodorakou E Tsoulfas G. Hepatic ischemia and reperfusion injury and trauma: current concepts. Arch Trauma Res. (2013) 2:63–70. doi: 10.5812/atr.12501, PMID: 24396796 PMC3876547

[4] Liu J Man K. Mechanistic insight and clinical implications of ischemia/reperfusion injury post liver transplantation. Cell Mol Gastroenterol Hepatol. (2023) 15:1463–74. doi: 10.1016/j.jcmgh.2023.03.003, PMID: 36940849 PMC10160787

[5] Liu XB Lo CM Cheng Q Ng KT Shao Y Li CX. Oval cells contribute to fibrogenesis of marginal liver grafts under stepwise regulation of aldose reductase and notch signaling. Theranostics. (2017) 7:4879–93. doi: 10.7150/thno.20085, PMID: 29187911 PMC5706107

[6] Wu K Ma L Xu T Cao J Zhou C Yu X. Transcription factor YY1 ameliorates liver ischemia-reperfusion injury through modulating the mir-181a-5p/ESR1/ERBB2 axis. Transplantation. (2023) 107:878–89. doi: 10.1097/TP.0000000000004356, PMID: 36413144

[7] Zhu S Wang X Chen H Zhu W Li X Cui R. Hippo (YAP)-autophagy axis protects against hepatic ischemia-reperfusion injury through JNK signaling. Chin Med J. (2024) 137:657–68. doi: 10.1097/CM9.0000000000002727, PMID: 37232477 PMC10950187

[8] Tang D Kang R Berghe TV Vandenabeele P Kroemer G. The molecular machinery of regulated cell death. Cell Res. (2019) 29:347–64. doi: 10.1038/s41422-019-0164-5, PMID: 30948788 PMC6796845

[9] Zhang M Xin W Yu Y Yang X Ma C Zhang H. Programmed death-ligand 1 triggers PASMCs pyroptosis and pulmonary vascular fibrosis in pulmonary hypertension. J Mol Cell Cardiol. (2020) 138:23–33. doi: 10.1016/j.yjmcc.2019.10.008, PMID: 31733200

[10] Liu ZY You QY Liu ZY Lin LC Yang JJ Tao H. m6A control programmed cell death in cardiac fibrosis. Life Sci. (2024) 353:122922. doi: 10.1016/j.lfs.2024.122922 39032691

[11] Song Z Gong Q Guo J. Pyroptosis: mechanisms and links with fibrosis. Cells. (2021) 10:3509. doi: 10.3390/cells10123509, PMID: 34944017 PMC8700428

[12] Tang SP Mao XL Chen YH Yan LL Ye LP Li SW. Reactive oxygen species induce fatty liver and ischemia-reperfusion injury by promoting inflammation and cell death. Front Immunol. (2022) 13:870239. doi: 10.3389/fimmu.2022.870239, PMID: 35572532 PMC9098816

[13] Xu Y Tang Y Lu J Zhang W Zhu Y Zhang S. PINK1-mediated mitophagy protects against hepatic ischemia/reperfusion injury by restraining NLRP3 inflammasome activation. Free Radic Biol Med. (2020) 160:871–86. doi: 10.1016/j.freeradbiomed.2020.09.015 32947010

[14] Luo S Luo R Deng G Huang F Lei Z. Programmed cell death, from liver ischemia-reperfusion injury perspective: an overview. Heliyon. (2024) 10:e32480. doi: 10.1016/j.heliyon.2024.e32480, PMID: 39040334 PMC11260932

[15] Luo L Mo G Huang D. Ferroptosis in hepatic ischemia-reperfusion injury: regulatory mechanisms and new methods for therapy (review). Mol Med Rep. (2021) 23:225. doi: 10.3892/mmr.2021.11864, PMID: 33495834

[16] Liu H Guo H Jian Z Cui H Fang J Zuo Z. Copper induces oxidative stress and apoptosis in the mouse liver. Oxidative Med Cell Longev. (2020) 2020:1–20. doi: 10.1155/2020/1359164, PMID: 32411316 PMC7201649

[17] El-Sisi AEE Sokar SS Shebl AM Mohamed DZ Abu-Risha SE. Octreotide and melatonin alleviate inflammasome-induced pyroptosis through inhibition of TLR4-NF-κb-NLRP3 pathway in hepatic ischemia/reperfusion injury. Toxicol Appl Pharmacol. (2021) 410:115340. doi: 10.1016/j.taap.2020.115340, PMID: 33264646

[18] Lv H Du Y Xin P Zhong Y Liu Z Jing Y. Identification of prevention marker associated with DNA replication in ovarian Cancer: expression of MCM2 protein and bioinformatics analysis. Int J Biol Macromol. (2024) 279:135079. doi: 10.1016/j.ijbiomac.2024.135079, PMID: 39187104

[19] Li A Zhang K Zhou J Li M Fan M Gao H. Bioinformatics and experimental approach identify lipocalin 2 as a diagnostic and prognostic Indicator for lung adenocarcinoma. Int J Biol Macromol. (2024) 272:132797. doi: 10.1016/j.ijbiomac.2024.132797, PMID: 38848833

[20] de Jonge J Kurian S Shaked A Reddy KR Hancock W Salomon DR. Unique early gene expression patterns in human adult-to-adult living donor liver grafts compared to deceased donor grafts. Am J Transplant. (2009) 9:758–72. doi: 10.1111/j.1600-6143.2009.02557.x, PMID: 19353763 PMC2734955

[21] Jassem W Xystrakis E Ghnewa YG Yuksel M Pop O Martinez-Llordella M. Normothermic machine perfusion (NMP) inhibits proinflammatory responses in the liver and promotes regeneration. Hepatology. (2019) 70:682–95. doi: 10.1002/hep.30475, PMID: 30561835

[22] Conti A Scala S D'Agostino P Alimenti E Morelli D Andria B. Wide gene expression profiling of ischemia-reperfusion injury in human liver transplantation. Liver Transpl. (2007) 13:99–113. doi: 10.1002/lt.20960, PMID: 17192907

[23] Leek JT Johnson WE Parker HS Jaffe AE Storey JD. The sva package for removing batch effects and other unwanted variation in high-throughput experiments. Bioinformatics (Oxford, England). (2012) 28:882–3. doi: 10.1093/bioinformatics/bts034, PMID: 22257669 PMC3307112

[24] Sosa RA Terry AQ Kaldas FM Jin YP Rossetti M Ito T. Disulfide high-mobility group box 1 drives ischemia-reperfusion injury in human liver transplantation. Hepatology. (2021) 73:1158–75. doi: 10.1002/hep.31324, PMID: 32426849 PMC8722704

[25] Wang S Wang R Hu D Zhang C Cao P Huang J. Machine learning reveals diverse cell death patterns in lung adenocarcinoma prognosis and therapy. NPJ Precis Oncol. (2024) 8:49. doi: 10.1038/s41698-024-00538-5, PMID: 38409471 PMC10897292

[26] Mustafa S Koran S AlOmair L. Insights into the role of matrix metalloproteinases in Cancer and its various therapeutic aspects: a review. Front Mol Biosci. (2022) 9:896099. doi: 10.3389/fmolb.2022.896099 36250005 PMC9557123

[27] Liu X Nie L Zhang Y Yan Y Wang C Colic M. Actin cytoskeleton vulnerability to disulfide stress mediates disulfidptosis. Nat Cell Biol. (2023) 25:404–14. doi: 10.1038/s41556-023-01091-2, PMID: 36747082 PMC10027392

[28] Zou Y Xie J Zheng S Liu W Tang Y Tian W. Leveraging diverse cell-death patterns to predict the prognosis and drug sensitivity of triple-negative breast Cancer patients after surgery. Int J Surg. (2022) 107:106936. doi: 10.1016/j.ijsu.2022.106936, PMID: 36341760

[29] Qin H Abulaiti A Maimaiti A Abulaiti Z Fan G Aili Y. Integrated machine learning survival framework develops a prognostic model based on inter-crosstalk definition of mitochondrial function and cell death patterns in a large multicenter cohort for lower-grade glioma. J Transl Med. (2023) 21:588. doi: 10.1186/s12967-023-04468-x, PMID: 37660060 PMC10474752

[30] Hanzelmann S Castelo R Guinney J. GSVA: gene set variation analysis for microarray and RNA-seq data. BMC Bioinformatics. (2013) 14:7. doi: 10.1186/1471-2105-14-7, PMID: 23323831 PMC3618321

[31] Yu G Wang L-G Han Y He Q-Y. ClusterProfiler: an R package for comparing biological themes among gene clusters. OMICS. (2012) 16:284–7. doi: 10.1089/omi.2011.0118, PMID: 22455463 PMC3339379

[32] Zhou Y Zhou B Pache L Chang M Khodabakhshi AH Tanaseichuk O. Metascape provides a biologist-oriented resource for the analysis of systems-level datasets. Nat Commun. (2019) 10:1523. doi: 10.1038/s41467-019-09234-6, PMID: 30944313 PMC6447622

[33] Chen B Sun X Huang H Feng C Chen W Wu D. An integrated machine learning framework for developing and validating a diagnostic model of major depressive disorder based on interstitial cystitis-related genes. J Affect Disord. (2024) 359:22–32. doi: 10.1016/j.jad.2024.05.061, PMID: 38754597

[34] Cui Y Zhang H Wang Z Gong B Al-Ward H Deng Y. Exploring the shared molecular mechanisms between systemic lupus erythematosus and primary Sjogren's syndrome based on integrated bioinformatics and single-cell RNA-seq analysis. Front Immunol. (2023) 14:1212330. doi: 10.3389/fimmu.2023.1212330, PMID: 37614232 PMC10442653

[35] Zhang Z Liu Y Huang D Huang Z. Single-cell WGCNA combined with transcriptome sequencing to study the molecular mechanisms of inflammation-related ferroptosis in myocardial ischemia-reperfusion injury. J Inflamm Res. (2024) 17:6203–27. doi: 10.2147/JIR.S476456, PMID: 39281774 PMC11397271

[36] Wang J. An R-based integrative tool for decoding human transcription factor-target interactions. Biomol Ther. (2024) 14:749. doi: 10.3390/biom14070749, PMID: 39062464 PMC11274450

[37] Zhang Q Liu W Zhang HM Xie GY Miao YR Xia M. hTFtarget: a comprehensive database for regulations of human transcription factors and their targets. Genomics Proteomics Bioinformatics. (2020) 18:120–8. doi: 10.1016/j.gpb.2019.09.006, PMID: 32858223 PMC7647694

[38] ENCODE Project Consortium. An integrated encyclopedia of DNA elements in the human genome. Nature. (2012) 489:57–74. doi: 10.1038/nature11247, PMID: 22955616 PMC3439153

[39] Rauluseviciute I Riudavets-Puig R Blanc-Mathieu R Castro-Mondragon JA Ferenc K Kumar V. JASPAR 2024: 20th anniversary of the open-access database of transcription factor binding profiles. Nucleic Acids Res. (2024) 52:D174–82. doi: 10.1093/nar/gkad1059 37962376 PMC10767809

[40] Kolmykov S Yevshin I Kulyashov M Sharipov R Kondrakhin Y Makeev VJ. GTRD: an integrated view of transcription regulation. Nucleic Acids Res. (2021) 49:D104–11. doi: 10.1093/nar/gkaa1057 33231677 PMC7778956

[41] Zou Z Ohta T Miura F Oki S. ChIP-atlas 2021 update: a data-mining suite for exploring epigenomic landscapes by fully integrating ChIP-seq, ATAC-seq and bisulfite-seq data. Nucleic Acids Res. (2022) 50:W175–82. doi: 10.1093/nar/gkac199, PMID: 35325188 PMC9252733

[42] Xiao L Chen J He X Zhang X Luo W. Whole-transcriptome sequencing revealed the ceRNA regulatory network during the proliferation and differentiation of goose myoblast. Poult Sci. (2024) 103:104173. doi: 10.1016/j.psj.2024.104173, PMID: 39153268 PMC11471125

[43] Zhong L Yang X Shang Y Yang Y Li J Liu S. Exploring the pathogenesis, biomarkers, and potential drugs for type 2 diabetes mellitus and acute pancreatitis through a comprehensive bioinformatic analysis. Front Endocrinol. (2024) 15:1405726. doi: 10.3389/fendo.2024.1405726, PMID: 39634181 PMC11614670

[44] Liu J Zhong L Zhang Y Ma J Xie T Chen X. Identification of novel biomarkers based on lipid metabolism-related molecular subtypes for moderately severe and severe acute pancreatitis. Lipids Health Dis. (2024) 23:1. doi: 10.1186/s12944-023-01972-3, PMID: 38169383 PMC10763093

[45] Masior L Grat M. Methods of attenuating ischemia-reperfusion injury in liver transplantation for hepatocellular carcinoma. Int J Mol Sci. (2021) 22:8229. doi: 10.3390/ijms22158229, PMID: 34360995 PMC8347959

[46] Eltzschig HK Eckle T. Ischemia and reperfusion--from mechanism to translation. Nat Med. (2011) 17:1391–401. doi: 10.1038/nm.2507 22064429 PMC3886192

[47] Zhai Y Petrowsky H Hong JC Busuttil RW Kupiec-Weglinski JW. Ischaemia-reperfusion injury in liver transplantation--from bench to bedside. Nat Rev Gastroenterol Hepatol. (2013) 10:79–89. doi: 10.1038/nrgastro.2012.225, PMID: 23229329 PMC3577927

[48] Chouchani ET Pell VR James AM Work LM Saeb-Parsy K Frezza C. A unifying mechanism for mitochondrial superoxide production during ischemia-reperfusion injury. Cell Metab. (2016) 23:254–63. doi: 10.1016/j.cmet.2015.12.009, PMID: 26777689

[49] Zhai Y Busuttil RW Kupiec-Weglinski JW. Liver ischemia and reperfusion injury: new insights into mechanisms of innate-adaptive immune-mediated tissue inflammation. Am J Transplant. (2011) 11:1563–9. doi: 10.1111/j.1600-6143.2011.03579.x, PMID: 21668640 PMC3658307

[50] Carroll PA Freie BW Mathsyaraja H Eisenman RN. The MYc transcription factor network: balancing metabolism, proliferation and oncogenesis. Front Med. (2018) 12:412–25. doi: 10.1007/s11684-018-0650-z, PMID: 30054853 PMC7358075

[51] Xu D Wang B Chen PP Wang YZ Miao NJ Yin F. c-Myc promotes tubular cell apoptosis in ischemia-reperfusion-induced renal injury by negatively regulating c-FLIP and enhancing FasL/Fas-mediated apoptosis pathway. Acta Pharmacol Sin. (2019) 40:1058–66. doi: 10.1038/s41401-018-0201-9, PMID: 30593588 PMC6786400

[52] Xiao Y Zheng S Duan N Li X Wen J. MicroRNA-26b-5p alleviates cerebral ischemia-reperfusion injury in rats via inhibiting the N-myc/PTEN axis by downregulating KLF10 expression. Hum Exp Toxicol. (2021) 40:1250–62. doi: 10.1177/0960327121991899 33559506

[53] Sun Z Shen L Sun X Tong G Sun D Han T. Variation of NDRG2 and c-Myc expression in rat heart during the acute stage of ischemia/reperfusion injury. Histochem Cell Biol. (2011) 135:27–35. doi: 10.1007/s00418-010-0776-9, PMID: 21193923

[54] Gnanaprakasam JNR Wang R. MYC in regulating immunity: metabolism and beyond. Genes (Basel). (2017) 8:88. doi: 10.3390/genes8030088, PMID: 28245597 PMC5368692

[55] Gnanaprakasam JNR Sherman JW Wang R. MYC and HIF in shaping immune response and immune metabolism. Cytokine Growth Factor Rev. (2017) 35:63–70. doi: 10.1016/j.cytogfr.2017.03.004 28363691

[56] Ji Y Wang Z Li Z Huang N Chen H Li B. Silencing IGF-II impairs c-Myc and N-ras expressions of SMMC-7721 cells via suppressing FAK/PI3K/AKT signaling pathway. Cytokine. (2017) 90:44–53. doi: 10.1016/j.cyto.2016.10.008, PMID: 27768959

[57] Zhang L Zhao D Wang Y Zhang W Zhang J Fan J. Focal adhesion kinase (FAK) inhibitor-defactinib suppresses the malignant progression of human esophageal squamous cell carcinoma (ESCC) cells via effective blockade of PI3K/AKT axis and downstream molecular network. Mol Carcinog. (2021) 60:113–24. doi: 10.1002/mc.23273, PMID: 33283357

[58] Wang C Sun Y Cong S Zhang F. Insulin-like growth Factor-1 promotes human uterine leiomyoma cell proliferation via PI3K/AKT/mTOR pathway. Cells Tissues Organs. (2023) 212:194–202. doi: 10.1159/000525186, PMID: 35605589

[59] De Vincenzo A Belli S Franco P Telesca M Iaccarino I Botti G. Paracrine recruitment and activation of fibroblasts by c-Myc expressing breast epithelial cells through the IGFs/IGF-1R axis. Int J Cancer. (2019) 145:2827–39. doi: 10.1002/ijc.32613, PMID: 31381136

