# Supplementary figures and images for: Identification of biomarkers associated with programmed cell death in liver ischemia–reperfusion injury: insights from machine learning frameworks and molecular docking in multiple cohorts
Source: Front Med (Lausanne). 2025 Mar 14;12:1501467. doi: 10.3389/fmed.2025.1501467 (PMC11949969; doi:10.3389/fmed.2025.1501467)

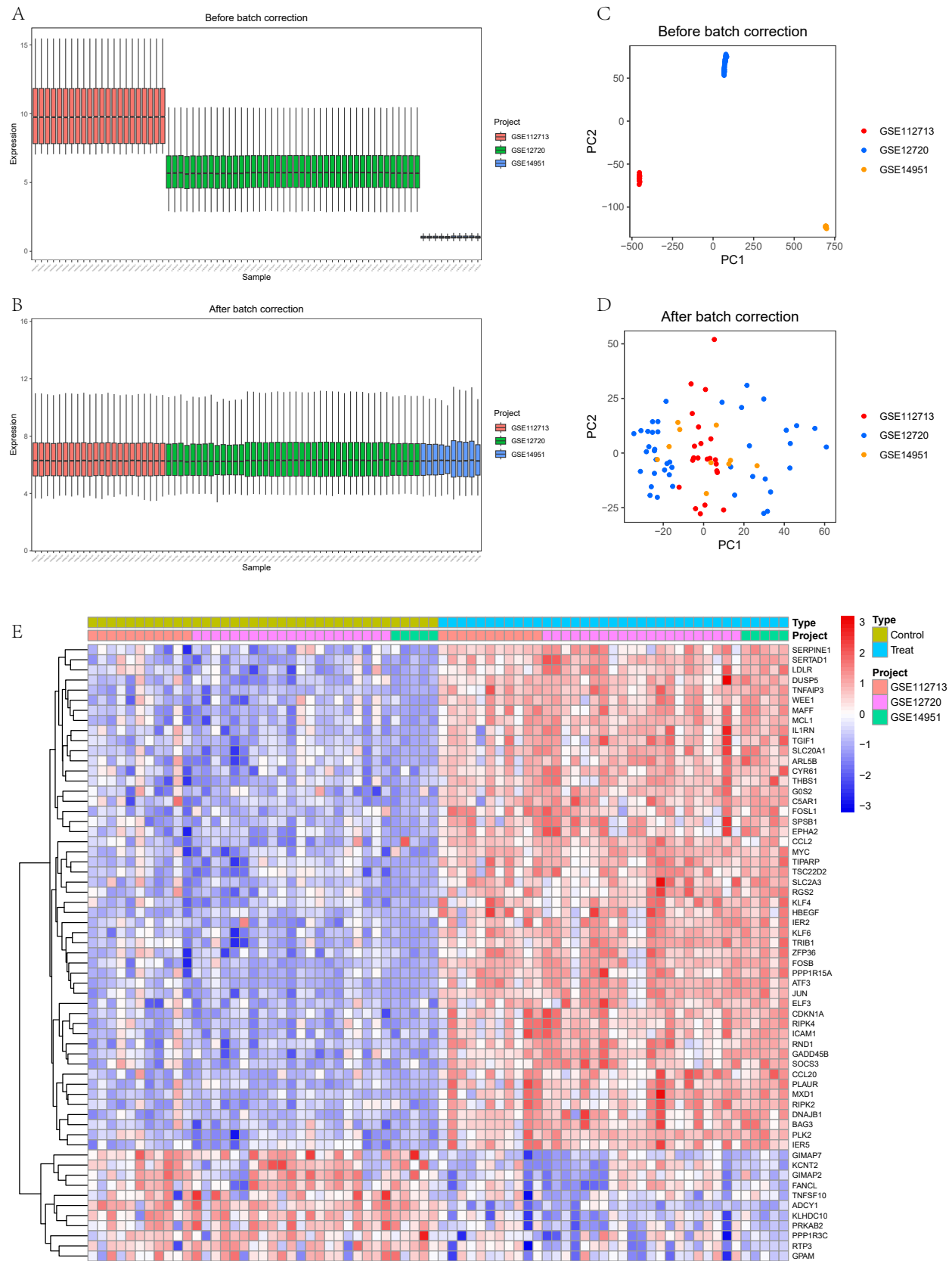

Supplement: Supplementary Figure S1 — (A,B) Box plot of expression level before and after merging of datasets; (C,D) PCA analysis before and after merging of datasets; (E) The heat map for DEGs in LIRI. [file Image_1.pdf]

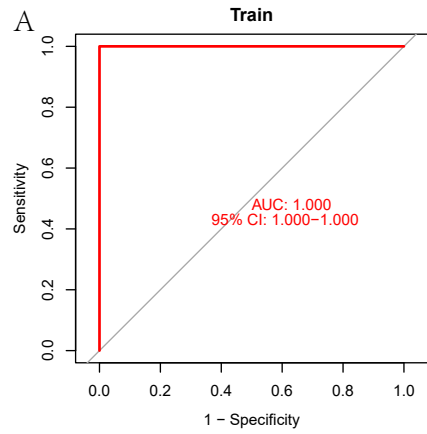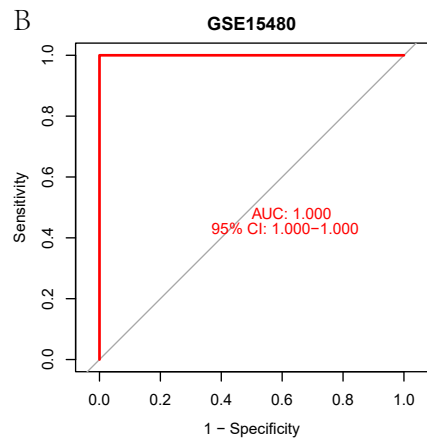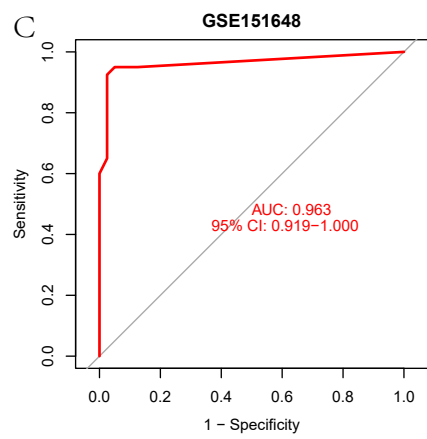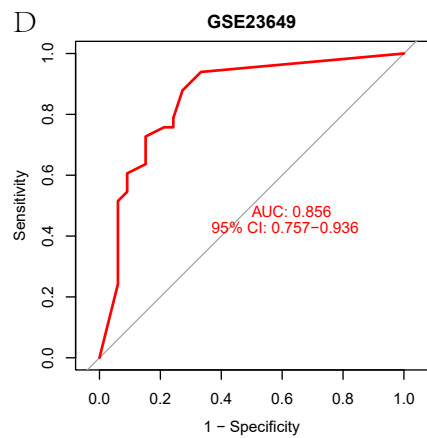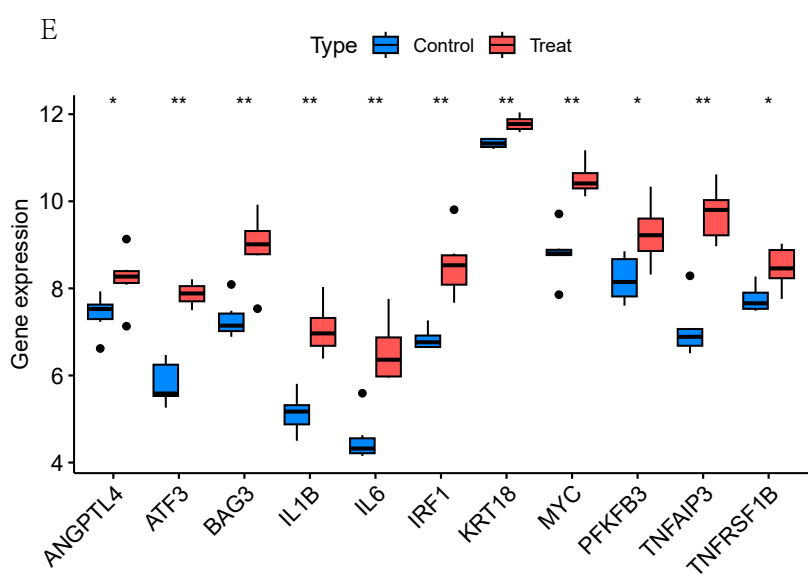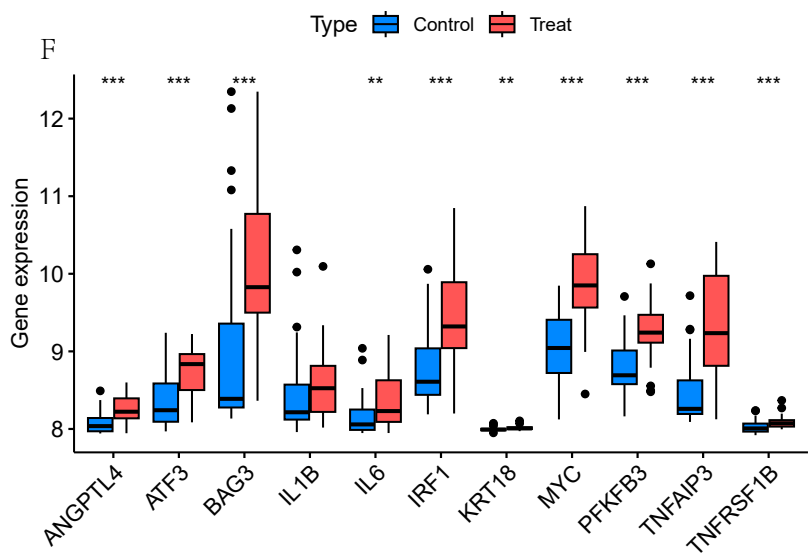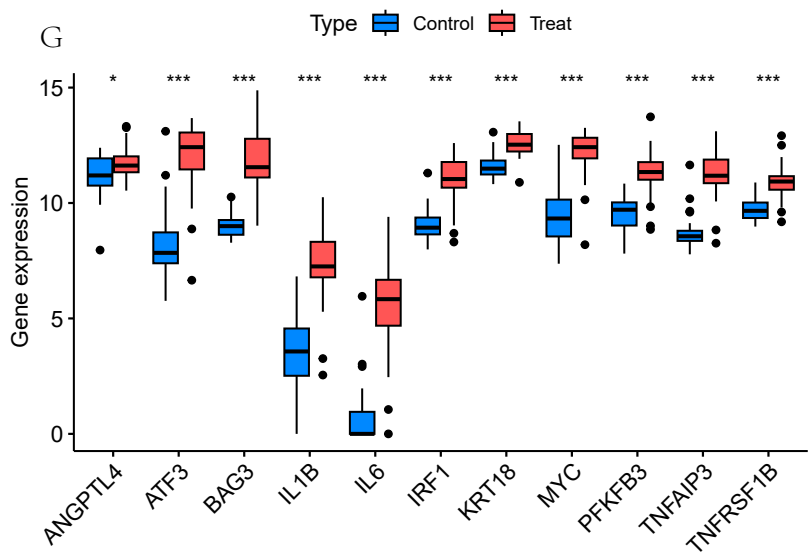

Supplement: Supplementary Figure S2 — (A–D) The ROC curves of our model in training and three test sets; (E–G) Box plots showed the expression difference of model genes between LIRI and control samples in test sets. [file Image_2.pdf]
