# Supplementary material for: Identification of biomarkers associated with programmed cell death in liver ischemia–reperfusion injury: insights from machine learning frameworks and molecular docking in multiple cohorts
Source: Front Med (Lausanne). 2025 Mar 14;12:1501467. doi: 10.3389/fmed.2025.1501467 (PMC11949969; doi:10.3389/fmed.2025.1501467)
Supplement: Supplementary Table S1 — List of 19 PCD-related genes. [file Table_1.pdf]

Apoptosis	Pyroptosis	Ferroptosis	Autophagy	Necroptosis	Cuproptosis	Parthanatos	Entoticcelldeath
AATF	BAK1	ABCC1	ABL1	GLUD1	NFE2L2	PARP	AMPK
ABL1	BAX	ACACA	ABL2	GLUD2	NLRP3	MIF	ATG5
ACAA2	CASP1	ACO1	ACER2	ALOX15	ATP7B	AIFM1	ATG7
ACKR3	CASP3	ACSF2	ADRA1A	FTH1	ATP7A	HSP70	BECN1
ACVR1	CASP4	ACSL1	ADRB2	PYG	SLC31A1	PAAN	CDC42
ACVR1B	CASP5	ACSL3	AKT1	CAPN1	FDX1	ARH3	CDH1
ADORA1	CASP6	ACSL4	AMBRA1	CASP1	LIAS	RNF146	CTNNA1
AEN	CASP8	ACSL5	ATF6	GLNA	LIPT1	ADPRHL2	CYBB
AGT	CASP9	ACSL6	ATG101	BAX	LIPT2	OGG1	MYH14
AGTR2	CHMP2A	AIFM2	ATG13	BCL2	DLD		PI3KC3
AIFM1	CHMP2B	AKR1C1	ATG14	FADD	DLAT		RHOA
AKT1	CHMP3	AKR1C2	ATG2A	RIPK1	PDHA1		RNF146
ANXA6	CHMP4A	AKR1C3	ATG2B	TNF	PDHB		ROCK
APAF1	CHMP4B	ALOX12	ATG5	TNFRSF1A	MTF1		RUBCN
APPL1	CHMP4C	ALOX15	ATG7	TRADD	GLS		UVRAG
AR	CHMP6	ALOX5	ATM	TRAF2	CDKN2A		
ARHGEF2	CHMP7	ATG5	ATP13A2	PPIA	DBT		
ARL6IP5	CYCS	ATG7	ATP6V0A1	CAPN2	GCSH		
ARMC10	ELANE	ATP5MC3	ATP6V0A2	HSP90A	DLST		
ARRB2	GPX4	BACH1	ATP6V0B	IL1A			
ASAH2	GSDMB	CARS	ATP6V0C	TNFSF6			
ATF3	GSDMC	CBS	ATP6V0D1	TNFRSF6			
ATF4	GSDMD	CD44	ATP6V0D2	CASP8			
ATM	GSDME	CHAC1	ATP6V0E1	JNK			
ATP2A1	GZMB	CISD1	ATP6V0E2	JAK2			
ATP2A3	HMGB1	CP	ATP6V1A	CAMK2			
ATP5IF1	IL18	CRYAB	ATP6V1B1	IL1B			
AVP	IL1A	CS	ATP6V1B2	IFNG			
BAD	IL1B	CYBB	ATP6V1C1	STAT3			
BAG3	IRF1	DPP4	ATP6V1C2	IRF9			
BAG5	IRF2	EMC2	ATP6V1D	TNFSF10			
BAG6	NLRC4	FADS2	ATP6V1E1	TNFRSF10A			
BAK1	NLRP1	FANCD2	ATP6V1E2	TNFRSF10B			
BAX	NLRP2	FDFT1	ATP6V1G1	CFLAR			
BBC3	NLRP3	FTH1	ATP6V1G2	XIAP			
BCAP31	NLRP6	FTL	ATP6V1H	BID			
BCL10	NLRP7	FTMT	AUP1	AIFM1			
BCL2	NOD1	G6PD	BAD	TRPM7			
BCL2A1	PLCG1	GCLC	BAG3	IFNAR1			
BCL2L1	PJVK	GCLM	BCL2	IFNAR2			
BCL2L10	PRKACA	GLS2	BCL2L11	IFNGR1			
BCL2L11	PYCARD	GOT1	BECN1	IFNGR2			
BCL2L12	SCAF11	GPX4	BMF	TLR3			
BCL2L14	TINAP	GSS	BNIP3	TIRP			
BCL2L2	TNF	HMGCR	BNIP3L	IFNA			
BCL3	TP53	HMOX1	BOK	IFNB			

BCLAF1	TP63	HSBP1	C9orf72	TRIF
BDKRB2	AIM2	HSPB1	CALCOCO2	VDAC1
BDNF	GSDMA	IREB2	CAMKK2	SLC25A4S
BECN1	IL6	KEAP1	CAPN1	PPID
BID	NOD2	LPCAT3	CAPNS1	CYLD
BIK	TIRAP	MAP1LC3A	CASP1	RIPK3
BIRC6		MAP1LC3B	CASP3	MLKL
BLOC1S2		MAP1LC3C	CDC37	TRAF5
BMF		MT1G	CDK5	TLR4
BMP4		NCOA4	CDK5R1	RBCK1
BMP5		NFE2L2	CHMP4A	HMGB1
BMPR1B		NFS1	CHMP4B	JAK1
BNIP3		NOX1	CISD2	JAK3
BNIP3L		NQO1	CLEC16A	TYK2
BOK		NRF2	CLN3	STAT1
BRCA1		OTUB1	CLU	STAT2
BRCA2		PCBP1	CPTP	STAT4
BRSK2		PCBP2	CSNK2A2	STAT5A
BTK		PEBP1	CTSA	STAT5B
CAAP1		PGD	CTTN	STAT6
CASP1		PHKG2	DAP	H2A
CASP10		PRNP	DAPK1	TNFAIP3
CASP12		PROM2	DAPK2	RNF31
CASP2		PTGS2	DAPK3	CHMP2A
CASP3		RPL8	DAPL1	CHMP2B
CASP4		SAT1	DCN	VPS24
CASP5		SAT2	DDIT3	CHMP4A
CASP8		SLC11A2	DDRGK1	CHMP4B
CASP8AP2		SLC1A5	DEPDC5	CHMP6
CASP9		SLC39A14	DEPP1	VPS4
CAV1		SLC39A8	DHRSX	CHMP1
CCAR2		SLC3A2	DNM1L	CHMP5
CCK		SLC40A1	DRAM1	SMPD1
CD14		SLC7A11	DRAM2	PYCARD
CD24		SQLE	EEF1A1	NLRP3
CD27		STEAP3	EEF1A2	ZBP1
CD28		TF	EIF2AK4	IL33
CD38		TFRC	EIF4G1	FTL
CD3E		TP53	EIF4G2	SQSTM1
CD44		VDAC2	ELAPOR1	VDAC2
CD5		VDAC3	EP300	VDAC3
CD70		ZEB1	EPM2A	CHMP7
CD74			ERCC4	PGAM5
CDIP1			ERN1	BIRC2
CDKN1A			EXOC1	BIRC3
CDKN2D			EXOC4	EIF2AK2
CEBPB			EXOC7	PLA2G4
CFLAR			EXOC8	DNM1L
CHAC1			FBXL2	SPATA2
CHCHD10			FBXO7	FAF1

CHEK2	FBXW7	SHARPIN
CIB1	FEZ1	NOX2
CIDEB	FEZ2	USP21
CLU	FLCN	PARP1
COA8	FOXK1	CHMP4C
COL2A1	FOXK2	
CRADD	FOXO1	
CREB3	FOXO3	
CREB3L1	FTH1	
CRH	FTL	
CRIP1	FYCO1	
CSF2	FZD5	
CSNK2A1	GAPDH	
CSNK2A2	GATA4	
CTH	GBA	
CTNNA1	GFAP	
CTSC	GNAI3	
CTTN	GOLGA2	
CUL1	GPR137	
CUL2	GPR137B	
CUL3	GPSM1	
CUL4A	GSK3A	
CUL5	GSK3B	
CX3CL1	HAX1	
CX3CR1	HDAC6	
CXCL12	HERC1	
CYLD	HGF	
CYP1B1	HIF1A	
DAB2IP	HMGB1	
DAP	HMOX1	
DAP3	HSP90AA1	
DAPK1	HSPA8	
DAPK2	HSPB1	
DAPK3	HSPB8	
DAPL1	HTR2B	
DAXX	HTRA2	
DBH	HTT	
DCC	HUWE1	
DDIAS	IFI16	
DDIT3	IFNG	
DDIT4	IKBKG	
DDX3X	IL10	
DDX47	IL10RA	
DDX5	IL4	
DEDD	IRGM	
DEDD2	ITPR1	
DELE1	KAT5	
DEPTOR	KAT8	
DIABLO	KDM4A	
DIDO1	KDR	

DNAJA1
DNAJC10
DNM1L
DPF2
DYRK2
E2F1
E2F2
EDA2R
EIF2AK3
ELL3
ENO1
EP300
EPHA2
EPO
ERBB3
ERCC6
ERN1
ERN2
ERO1A
ERP29
EYA1
EYA2
EYA3
EYA4
FADD
FAF1
FAIM
FAIM2
FAM162A
FAS
FASLG
FASTK
FBH1
FBXW7
FEM1B
FGA
FGB
FGF10
FGFR1
FGFR3
FGG
FHIT
FIGNL1
FIS1
FNIP2
FXN
FYN
FZD9
G0S2
GABARAP
GATA1
KEAP1
KIF25
KLHL22
KLHL3
LACRT
LAMP1
LAMP2
LAMP3
LAMTOR1
LAMTOR2
LAMTOR3
LAMTOR4
LAMTOR5
LARP1
LEP
LEPR
LGALS8
LRRK2
LRSAM1
LZTS1
MAP1LC3A
MAP1LC3B
MAP1LC3C
MAP3K7
MAPK15
MAPK3
MAPK8
MAPT
MCL1
MEFV
MET
MFN2
MFSD8
MID2
MIR199A1
MIRLET7B
MLST8
MT3
MTCL1
MTDH
MTM1
MTMR3
MTMR4
MTMR8
MTMR9
MTOR
NCOA4
NEDD4
NLRP6
NOD1
NOD2

GATA4
GCLM
GDNF
GFRAL
GGCT
GHITM
GNAI2
GNAI3
GPER1
GPX1
GRINA
GSDME
GSK3A
GSK3B
GSKIP
GSTP1
GZMB
HDAC1
HERPUD1
HGF
HIC1
HIF1A
HINT1
HIP1
HIP1R
HIPK1
HIPK2
HMGB2
HMOX1
HNRNPK
HRAS
HRK
HSPA1A
HSPA1B
HSPB1
HTRA2
HTT
HYAL2
HYOU1
ICAM1
IFI16
IFI27
IFI27L1
IFI27L2
IFI6
IFNB1
IFNG
IGF1
IKBKE
IL12A
IL19
NPC1
NPRL2
NRBP2
NUPR1
OPTN
ORMDL3
OSBPL7
PAFAH1B2
PARK7
PHB2
PHF23
PIK3C2A
PIK3C3
PIK3CA
PIK3CB
PIK3R2
PIM2
PINK1
PIP4K2A
PIP4K2B
PIP4K2C
PJVK
PLEKHF1
PLK2
PLK3
POLDIP2
PRKAA1
PRKAA2
PRKAB1
PRKAB2
PRKACA
PRKAG1
PRKAG2
PRKAG3
PRKD1
PRKN
PSAP
PTPN22
PYCARD
QSOX1
RAB39B
RAB3GAP1
RAB3GAP2
RAB7A
RAB8A
RALB
RASIP1
RB1CC1
RETREG1
RETREG3
RHEB

IL1A
IL1B
IL2
IL20RA
IL33
IL4
IL6R
IL7
INCA1
ING2
ING5
INHBA
INHBB
INS
ITGA6
ITGAM
ITGAV
ITM2C
ITPR1
ITPRIP
IVNS1ABP
JAK2
JMY
JUN
KDM1A
KITLG
KRT18
KRT8
LCK
LGALS12
LGALS3
LRRK2
LTBR
LY96
MADD
MAEL
MAGEA3
MAP2K5
MAP3K5
MAPK7
MAPK8
MAPK8IP1
MAPK8IP2
MAPK9
MARCHF7
MAZ
MCL1
MDM2
MELK
MFF
MIF
RIPK2
RMC1
RNF152
RNF41
RNF5
ROCK1
RPTOR
RRAGA
RRAGB
RRAGC
RRAGD
RUBCN
RUFY4
SCFD1
SCOC
SEC22B
SESN1
SESN2
SESN3
SH3BP4
SH3GLB1
SIRT1
SIRT2
SLC38A9
SMCR8
SMG1
SNCA
SNRNP70
SNX32
SNX5
SNX6
SOGA1
SOGA3
SPTLC1
SPTLC2
SQSTM1
SREBF1
SREBF2
STAT3
STBD1
STING1
STK11
STUB1
SUPT5H
SVIP
SYNPO2
TAB2
TAB3
TBC1D14
TBC1D25
TBK1

MIR132	TEX264
MIR15A	TFEB
MIR16-1	TICAM1
MIR17	TIGAR
MIR198	TLK2
MIR21	TMEM150A
MIR210	TMEM150B
MIR221	TMEM150C
MIR222	TMEM39A
MIR26B	TMEM39B
MIR27B	TMEM59
MIR449A	TOMM7
MKNK2	TP53
MLH1	TP53INP1
MLLT11	TP53INP2
MMP9	TPCN1
MNT	TPCN2
MOAP1	TREM2
MPV17L	TRIB3
MSH2	TRIM13
MSH6	TRIM14
MSX1	TRIM21
MUC1	TRIM22
MUL1	TRIM27
MYBBP1A	TRIM34
NACC2	TRIM38
NANOS3	TRIM5
NBN	TRIM6
NCK1	TRIM65
NCK2	TRIM68
NDUFA13	TRIM8
NDUFS3	TRIML1
NFATC4	TRIML2
NFE2L2	TSC1
NGF	TSC2
NGFR	TSPO
NKX3-1	UBA5
NLE1	UBQLN1
NME5	UBQLN2
NMT1	UBQLN4
NOC2L	UCHL1
NOG	UFC1
NOL3	UFL1
NONO	UFM1
NOS3	ULK1
NOX1	USP10
NR4A2	USP13
NUPR1	USP30

OPA1
P2RX4
P2RX7
P4HB
PAK2
PAK5
PARK7
PARP1
PARP2
PAWR
PCGF2
PDCD10
PDCD5
PDCD6
PDIA3
PDK1
PDK2
PDPK1
PDX1
PEA15
PELI3
PERP
PF4
PHIP
PHLDA3
PIAS4
PIDD1
PIH1D1
PIK3R1
PINK1
PLAGL2
PLAUR
PLEKHF1
PLSCR3
PMAIP1
PML
POLB
POU4F1
POU4F2
PPARD
PPIA
PPIF
PPM1F
PPP1CA
PPP1R13B
PPP1R15A
PPP2R1B
PPP3CC
PPP3R1
PRDX2
PRELID1
USP33
USP36
UVRAG
VDAC1
VPS13C
VPS13D
VPS26A
VPS26B
VPS35
WAC
WASHC1
WDFY3
WDR24
WDR41
WDR6
WDR81
WIPI2
ZC3H12A
ZKSCAN3
ZMPSTE24

PRKCA
PRKCD
PRKDC
PRKN
PRKRA
PRODH
PSEN1
PSMD10
PSME3
PTEN
PTGIS
PTH
PTPMT1
PTPN1
PTPN2
PTPRC
PTTG1IP
PYCARD
QARS1
RACK1
RAF1
RB1
RB1CC1
RBCK1
RELA
RET
RFFL
RHOT1
RHOT2
RIPK1
RIPK3
RNF183
RNF186
RNF34
RNF41
RPL11
RPL26
RPS27L
RPS3
RPS6KB1
RPS7
RRP8
RTKN2
RTL10
S100A8
S100A9
SCG2
SCN2A
SCRT2
SELENOK
SELENOS

SENP1
SEPTIN4
SERINC3
SERPINE1
SFN
SFPQ
SFRP1
SFRP2
SGMS1
SGPL1
SGPP1
SH3RF1
SHH
SHISA5
SIAH1
SIAH2
SIRT1
SIVA1
SKIL
SLC25A5
SLC35F6
SLC9A3R1
SMAD3
SNAI1
SNAI2
SNW1
SOD1
SOD2
SORT1
SP100
SRC
SRPX
SST
SSTR3
ST20
STK11
STK24
STK25
STK3
STK4
STRADB
STX4
STYXL1
SYVN1
TAF9
TAF9B
TCF7L2
TERT
TFDP1
TFDP2
TFPT

TGFB1
TGFB2
TGFBR1
THBS1
TICAM1
TICAM2
TIMM50
TIMP3
TLR3
TLR4
TM2D1
TMBIM1
TMBIM6
TMC8
TMEM102
TMEM109
TMEM117
TMEM14A
TMEM161
A
TNF
TNFAIP3
TNFRSF10
A
TNFRSF10
B
TNFRSF10
C
TNFRSF12
A
TNFRSF1A
TNFRSF1B
TNFRSF25
TNFSF10
TNFSF12
TOPORS
TP53
TP53BP2
TP63
TP73
TPD52L1
TPT1
TRADD
TRAF1
TRAF2
TRAF7
TRAP1
TRIAP1
TRIB3
TRIM32
TRIM39
TXNDC12
TYROBP

UACA
UBB
UBE2K
UBE4B
UBQLN1
UMOD
UNC5B
URI1
USP28
USP47
VDAC2
VNN1
WDR35
WNT4
WWOX
XBP1
YAP1
YBX3
YWHAB
YWHAE
YWHAG
YWHAH
YWHAQ
YWHAZ
ZC3HC1
ZDHHC3
ZMYND11
ZNF205
ZNF385A
ZNF385B
ZNF622
ZSWIM2

Netoticcelldeath	Lysosome-dependentcelldeath	Alkaliptosis	Oxeiptosis	NETosis	Immunogenic cell death
ELANE	ABCA2	IKBKB	PGAM5	MYD88	ATG5
MMP1	ABCB9	NFKB1	KEAP1	TLR2	BAX
MPO	ACP2	CA9	AIFM1	PAD4	CALR
CAMP	ACP5	CHUK	NRF2	PRKCA	CASP1
PADI4	ADGRE2	IKBKG	AIRE	PKCB	CASP8
EIPA	AGA	NFKB1A		PRKCZ	CD4
NCX1	AP1B1	RELA		NOX3	CD8A
MIA	AP1G1			NOX4	CD8B
	AP1M1			NOX1	CXCR3
	AP1M2			CTSG	EIF2AK3
	AP1S1			PRTN3	ENTPD1
	AP1S2			ELANE	FOXP3
	AP1S3			MPO	HMGB1
	AP3B1			GSDMD	HSP90AA1
	AP3B2			IL1B	IFNA1
	AP3D1			CXCL1	IFNB1
	AP3M1			PLA2G7	IFNG
	AP3M2			CXCL8	IFNGR1
	AP3S1			CDK6	IL10
	AP3S2			HMGB1	IL17A
	AP4B1			MMP9	IL17RA
	AP4E1			AGER	IL1B
	AP4M1			CSF3	IL1R1
	AP4S1			TGFB1	IL6
	ARF1				LY96
	ARL8B				MYD88
	ARSA				NLRP3
	ARSB				NT5E
	ARSG				P2RX7
	ASAH1				PDIA3
	ATP10B				PIK3CA
	ATP13A2				PRF1
	ATP6AP1				TLR4
	ATP6V0A1				TNF
	ATP6V0A2				
	ATP6V0A4				
	ATP6V0B				
	ATP6V0C				
	ATP6V0D1				
	ATP6V0D2				
	ATP6V1H				
	BLK				
	BLOC1S1				
	BLOC1S2				
	BORCS5				
	BORCS6				

BTK
C12orf4
CBL
CD164
CD300A
CD63
CD68
CD84
CHGA
CLN3
CLN5
CLNK
CLTA
CLTB
CLTC
CLTCL1
CLU
CPLX2
CTNS
CTSA
CTSB
CTSC
CTSD
CTSE
CTSF
CTSG
CTSH
CTSK
CTSL
CTSO
CTSS
CTSV
CTSW
CTSZ
DEF8
DNASE2
DNASE2B
ENTPD4
FAM98A
FER
FES
FGR
FLCN
FOXF1
FTH1
FTL
FUCA1
GAA
GAB2
GALC

GALNS
GATA2
GBA
GCC2
GGA1
GGA2
GGA3
GLA
GLB1
GM2A
GNPTAB
GNPTG
GNS
GUSB
HDAC6
HEXA
HEXB
HGS
HGSNAT
HMOX1
HPS6
HSPA8
HYAL1
IDS
IDUA
IGF2R
IL13
IL13RA2
IL4
IL4R
KIF1B
KIT
KXD1
LAMP1
LAMP2
LAMP3
LAMTOR1
LAPTM4A
LAPTM4B
LAPTM5
LAT
LAT2
LGALS9
LGMN
LIPA
LRRK2
LYN
M6PR
MAN2B1
MANBA

MAP1LC3A
MAP6
MCOLN1
MFSD8
MILR1
MRGPRX2
MT3
MYH9
NAGA
NAGLU
NAGPA
NAPSA
NCOA4
NDEL1
NEDD4
NEU1
NPC1
NPC2
NR4A3
PDPK1
PIK3C3
PIK3CD
PIK3CG
PIP4K2A
PIP4K2B
PIP4P1
PLA2G15
PLA2G3
PLEKHM1
PLEKHM2
PPT1
PPT2
PSAP
PSAPL1
PTGDR
PTGDS
RAB34
RAB3A
RAB7A
RAC2
RUBCNL
S100A13
SCARB2
SGSH
SLC11A1
SLC11A2
SLC17A5
SMPD1
SNAP23
SNAPIN
SNX16

SNX4
SORL1
SORT1
SPAG9
SPHK2
SQSTM1
STXBP1
STXBP2
SUMF1
SYK
SYTL4
TCIRG1
TFEB
TMEM106B
TPP1
UNC13D
VAMP7
VAMP8
VPS33A
VPS33B
VPS4A
WASH3P
ZFYVE16

**Anoikis**	**Paraptosis**	**Methuosis**	**Entosis**	**Disufidptosis**
BRMS1	CAMK2B	CSNK2A1	AR	GYS1
PTK2	PRKACG	RAC1	TP53	NDUFS1
NTRK2	MARK4	ARF6	ROCK1	OXSM
BCL2L11	SSTR5	GIT1	PCK2	LRPPRC
SRC	TAAR5	MTOR	TNFSF10	NDUFA11
CEACAM6	USP10	MET	MTUS2	NUBPL
CAV1	PRKAG3	PFKFB3	AURKA	NCKAP1
AKT1	HACD2	MIR199A1	KIF2C	RPN1
ITGB1	NT5C		RHOA	SLC3A2
CEACAM5	INSRR		MTOR	SLC7A11
EGFR	SSTR3		EZR	FLNA
BCL2	TAAR9		GZMB	FLNB
CASP8	HSPB8		MRTFA	MYH9
SIK1	PLPP2		PTK2	TLN1
PTRH2	G6PC2		DIAPH1	ACTB
STAT3	GUCY2EP		LPAR2	MYL6
TLE1	CDK4		MAP1LC3A	MYH10
DAPK2	RGR		PIKFYVE	CAPZB
CTNNB1	ADGRG1		CTTN	DSTN
ZNF304	UQCRC1		MCOLN1	IQGAP1
MAPK1	TNK2		FOXO1	ACTN4
BMF	RNF181		CTNNA1	PDLIM1
ITGA5	MKNK2		CXCL8	CD2AP
TP53	UBE2U			INF2
MCL1	MYLK			
BCL2L1	CTDSP2			
CASP3	LCK			
CDH1	GPR15			
BAD	ATP23			
PIK3CA	LPAR1			
PAK1	PI4KB			
ITGAV	DSTYK			
FN1	CFD			
MAPK3	PPP3CA			
PTGS2	CCR4			
BAX	PRAG1			
BCAR1	CDKN3			
PTEN	GPR153			
ERBB2	DDIT3			
ANGPTL4	MAPK8			
PDK4	MAP2K2			
CYCS	MAPK1			
BRAF	MAPK14			
YAP1	IGF1R			
ANKRD13C	PDCD6IP			
ITGA2	CASP9			

ANXA5	ERN1
BIRC5	ATF6
MTOR	XBP1
TIMP1	AKT1
BDNF	EIF2S1
CSPG4	HSPA5
BSG	CASP4
AKT2	CASP3
STK11	CASP7
IGF1	ITPR3
IGF1R	RYR1
ITGA6	RYR2
ILK	MCU
CFLAR	TNFRSF19
RHOA	PDCD5
HIF1A	CSF1
DAP3	TP53
MYBBP1A	NFKB1
TLE5	PEBP1
ITGA3	PHB
PTK2B	
CCND1	
CTTN	
CALR	
ATF4	
CDCP1	
PLAUR	
SKP2	
CHEK2	
HGF	
E2F1	
EGF	
PIK3CG	
ITGB4	
DAPK1	
MAPK8	
PIK3R1	
PIK3R3	
MAP2K1	
CXCL12	
LGALS3	
FBXW7-AS1	
BAK1	
ABHD4	
CD44	
ITGA4	
FADD	
PHLDA2	
TGFB1	
HMCN1	

MMP2
CEBPB
CEMIP
CDKN3
CBL
CASP9
SFN
MTDH
PRKCA
TNFRSF10
B
CXCL8
MIR200C
AR
CDKN2A
CPT1A
PIK3CB
CLDN1
MIR204
MIR26A1
CDKN1A
CDKN1B
KLF12
NTRK1
PLAU
MYC
SMAD4
PLK1
MUC1
LGALS1
PYCARD
SESN2
ITGB3
KRAS
THBS1
BID
HRAS
CDK11B
CDK11A
XIAP
PPARG
IL6
MIR145
CCR7
MSLN
RAC1
GRHL2
BIRC3
NOTCH1
RHOG
CCAR2

NQO1
MMP13
FAS
MTA1
MYO5A
EDA2R
CCN6
MMP9
ABL1
MAPK11
SOD2
PTHLH
PDGFB
GLI2
EZH2
RIPK1
CXCR4
HMGA1
SIK2
TNFSF10
ANGPTL2
S100A4
NTF3
ETV4
MIR21
MIR124-1
HTRA1
LATS1
CEACAM3
EIF2AK3
LAMC2
LAMA3
LAMB3
CDH2
CSNK2A1
EDIL3
ZEB2
TLN1
EPHA2
SIRT3
OLFM3
CLU
SPINK1
CPEB2
NAT1
TSG101
MIR200A
MIR6744
SERPINA1
AKT3
RELA

TNFRSF1A
FASLG
AFP
ITGA8
NOX4
PBK
SATB1
CD63
EEF1A1
LTB4R2
MAVS
HRC
CCN2
RHOB
PPP1R13B
PLG
MET
RAF1
PARP1
PRKCQ
BRCA2
RB1
SP1
HAVCR2
DOCK1
VTN
INHBB
PDCD4
PRPF4B
RANBP9
SESN1
SESN3
CD24
ZBTB7A
MIR141
ELANE
KDR
MDM2
NFE2L2
ZEB1
KL
PRKCI
CRYAB
EPHB6
FGF2
HK2
LTF
IQGAP1
MGAT5
SDCBP
ABHD2

SPIB
TRIM31
MIR1827
PDGFRB
PLAT
TLR3
NRAS
ROCK1
PAK4
VEGFA
CASP10
PIN1
IL1RAP
UBE2C
YWHAZ
TWIST1
BMP6
BNIP3L
ELK1
KDM3A
PRDX4
BNIP3
LMO3
ZNF32
MIR200B
MIR525
MIR363
TUBB3
HSP90B1
SLC2A1
HMOX1
PTPN11
PRKACA
PAK3
CD36
PIK3R2
PPP2CA
CASP6
CDH3
EEF2K
LRP1
PAK2
PTK6
LPAR1
TCF7L2
CEACAM1
GDF2
GLO1
IL17A
RBL2
SIRPA

TRAF2
ADCY10
VPS37A
TNFRSF12
A
APOBEC3
G
BAG1
COL13A1
MNX1
RAD9A
IFI27
MEGF11
ITPRIP
BCL2L15
SNAI2
PTPN1
NOTCH3
GLUD1
SIRT1
FASN
MYH9
RPS6KB1
TPM1
PPP2R1A
COL4A2
CTNND1
CD151
MMP11
ARHGEF7
PPP2R2A
SEMA7A
PPP2R5A
BST2
CCN1
PPP2R2D
CCDC178
MIR10A
MIR30B
MIR30C1
SHC1

AATF
ABCA2
ABCB9
ABCC1
ABHD2
ABHD4
ABL1
ABL2
ACAA2
ACACA
ACER2
ACKR3
ACO1
ACP2
ACP5
ACSF2
ACSL1
ACSL3
ACSL4
ACSL5
ACSL6
ACTB
ACTN4
ACVR1
ACVR1B
ADCY10
ADGRE2
ADGRG1
ADORA1
ADPRHL2
ADRA1A
ADRB2
AEN
AFP
AGA
AGER
AGT
AGTR2
AIFM1
AIFM2
AIM2
AIRE
AKR1C1
AKR1C2
AKR1C3
AKT1
AKT2
AKT3
ALOX12
ALOX15
ALOX5

AMBRA1
AMPK
ANGPTL2
ANGPTL4
ANKRD13
C
ANXA5
ANXA6
AP1B1
AP1G1
AP1M1
AP1M2
AP1S1
AP1S2
AP1S3
AP3B1
AP3B2
AP3D1
AP3M1
AP3M2
AP3S1
AP3S2
AP4B1
AP4E1
AP4M1
AP4S1
APAF1
APOBEC3
G
APPL1
AR
ARF1
ARF6
ARH3
ARHGEF2
ARHGEF7
ARL6IP5
ARL8B
ARMC10
ARRB2
ARSA
ARSB
ARSG
ASAH1
ASAH2
ATF3
ATF4
ATF6
ATG101
ATG13
ATG14
ATG2A

ATG2B
ATG5
ATG7
ATM
ATP10B
ATP13A2
ATP23
ATP2A1
ATP2A3
ATP5IF1
ATP5MC3
ATP6AP1
ATP6V0A1
ATP6V0A2
ATP6V0A4
ATP6V0B
ATP6V0C
ATP6V0D1
ATP6V0D2
ATP6V0E1
ATP6V0E2
ATP6V1A
ATP6V1B1
ATP6V1B2
ATP6V1C1
ATP6V1C2
ATP6V1D
ATP6V1E1
ATP6V1E2
ATP6V1G1
ATP6V1G2
ATP6V1H
ATP7A
ATP7B
AUP1
AURKA
AVP
BACH1
BAD
BAG1
BAG3
BAG5
BAG6
BAK1
BAX
BBC3
BCAP31
BCAR1
BCL10
BCL2
BCL2A1

BCL2L1
BCL2L10
BCL2L11
BCL2L12
BCL2L14
BCL2L15
BCL2L2
BCL3
BCLAF1
BDKRB2
BDNF
BECN1
BID
BIK
BIRC2
BIRC3
BIRC5
BIRC6
BLK
BLOC1S1
BLOC1S2
BMF
BMP4
BMP5
BMP6
BMPR1B
BNIP3
BNIP3L
BOK
BORCS5
BORCS6
BRAF
BRCA1
BRCA2
BRMS1
BRSK2
BSG
BST2
BTK
C12orf4
C9orf72
CA9
CAAP1
CALCOCO
2
CALR
CAMK2
CAMK2B
CAMKK2
CAMP
CAPN1

CAPN2
CAPNS1
CAPZB
CARS
CASP1
CASP10
CASP12
CASP2
CASP3
CASP4
CASP5
CASP6
CASP7
CASP8
CASP8AP2
CASP9
CAV1
CBL
CBS
CCAR2
CCDC178
CCK
CCN1
CCN2
CCN6
CCND1
CCR4
CCR7
CD14
CD151
CD164
CD24
CD27
CD28
CD2AP
CD300A
CD36
CD38
CD3E
CD4
CD44
CD5
CD63
CD68
CD70
CD74
CD84
CD8A
CD8B
CDC37
CDC42

CDCP1
CDH1
CDH2
CDH3
CDIP1
CDK11A
CDK11B
CDK4
CDK5
CDK5R1
CDK6
CDKN1A
CDKN1B
CDKN2A
CDKN2D
CDKN3
CEACAM1
CEACAM3
CEACAM5
CEACAM6
CEBPB
CEMIP
CFD
CFLAR
CHAC1
CHCHD10
CHEK2
CHGA
CHMP1
CHMP2A
CHMP2B
CHMP3
CHMP4A
CHMP4B
CHMP4C
CHMP5
CHMP6
CHMP7
CHUK
CIB1
CIDEB
CISD1
CISD2
CLDN1
CLEC16A
CLN3
CLN5
CLNK
CLTA
CLTB
CLTC

CLTCL1
CLU
COA8
COL13A1
COL2A1
COL4A2
CP
CPEB2
CPLX2
CPT1A
CPTP
CRADD
CREB3
CREB3L1
CRH
CRIP1
CRYAB
CS
CSF1
CSF2
CSF3
CSNK2A1
CSNK2A2
CSPG4
CTDSP2
CTH
CTNNA1
CTNNB1
CTNND1
CTNS
CTSA
CTSB
CTSC
CTSD
CTSE
CTSF
CTSG
CTSH
CTSK
CTSL
CTSO
CTSS
CTSV
CTSW
CTSZ
CTTN
CUL1
CUL2
CUL3
CUL4A
CUL5

CX3CL1
CX3CR1
CXCL1
CXCL12
CXCL8
CXCR3
CXCR4
CYBB
CYCS
CYLD
CYP1B1
DAB2IP
DAP
DAP3
DAPK1
DAPK2
DAPK3
DAPL1
DAXX
DBH
DBT
DCC
DCN
DDIAS
DDIT3
DDIT4
DDRGK1
DDX3X
DDX47
DDX5
DEDD
DEDD2
DEF8
DELE1
DEPDC5
DEPP1
DEPTOR
DHRSX
DIABLO
DIAPH1
DIDO1
DLAT
DLD
DLST
DNAJA1
DNAJC10
DNASE2
DNASE2B
DNM1L
DOCK1
DPF2

DPP4
DRAM1
DRAM2
DSTN
DSTYK
DYRK2
E2F1
E2F2
EDA2R
EDIL3
EEF1A1
EEF1A2
EEF2K
EGF
EGFR
EIF2AK2
EIF2AK3
EIF2AK4
EIF2S1
EIF4G1
EIF4G2
EIPA
ELANE
ELAPOR1
ELK1
ELL3
EMC2
ENO1
ENTPD1
ENTPD4
EP300
EPHA2
EPHB6
EPM2A
EPO
ERBB2
ERBB3
ERCC4
ERCC6
ERN1
ERN2
ERO1A
ERP29
ETV4
EXOC1
EXOC4
EXOC7
EXOC8
EYA1
EYA2
EYA3

EYA4
EZH2
EZR
FADD
FADS2
FAF1
FAIM
FAIM2
FAM162A
FAM98A
FANCD2
FAS
FASLG
FASN
FASTK
FBH1
FBXL2
FBXO7
FBXW7
FBXW7-
AS1
FDFT1
FDX1
FEM1B
FER
FES
FEZ1
FEZ2
FGA
FGB
FGF10
FGF2
FGFR1
FGFR3
FGG
FGR
FHIT
FIGNL1
FIS1
FLCN
FLNA
FLNB
FN1
FNIP2
FOXF1
FOXK1
FOXK2
FOXO1
FOXO3
FOXP3
FTH1

FTL
FTMT
FUCA1
FXN
FYCO1
FYN
FZD5
FZD9
G0S2
G6PC2
G6PD
GAA
GAB2
GABARAP
GALC
GALNS
GAPDH
GATA1
GATA2
GATA4
GBA
GCC2
GCLC
GCLM
GCSH
GDF2
GDNF
GFAP
GFRAL
GGA1
GGA2
GGA3
GGCT
GHITM
GIT1
GLA
GLB1
GLI2
GLNA
GLO1
GLS
GLS2
GLUD1
GLUD2
GM2A
GNAI2
GNAI3
GNPTAB
GNPTG
GNS
GOLGA2

GOT1
GPER1
GPR137
GPR137B
GPR15
GPR153
GPSM1
GPX1
GPX4
GRHL2
GRINA
GSDMA
GSDMB
GSDMC
GSDMD
GSDME
GSK3A
GSK3B
GSKIP
GSS
GSTP1
GUCY2EP
GUSB
GYS1
GZMB
H2A
HACD2
HAVCR2
HAX1
HDAC1
HDAC6
HERC1
HERPUD1
HEXA
HEXB
HGF
HGS
HGSNAT
HIC1
HIF1A
HINT1
HIP1
HIP1R
HIPK1
HIPK2
HK2
HMCN1
HMGA1
HMGB1
HMGB2
HMGCR

HMOX1
HNRNPK
HPS6
HRAS
HRC
HRK
HSBP1
HSP70
HSP90A
HSP90AA1
HSP90B1
HSPA1A
HSPA1B
HSPA5
HSPA8
HSPB1
HSPB8
HTR2B
HTRA1
HTRA2
HTT
HUWE1
HYAL1
HYAL2
HYOU1
ICAM1
IDS
IDUA
IFI16
IFI27
IFI27L1
IFI27L2
IFI6
IFNA
IFNA1
IFNAR1
IFNAR2
IFNB
IFNB1
IFNG
IFNGR1
IFNGR2
IGF1
IGF1R
IGF2R
IKBKB
IKBKE
IKBKG
IL10
IL10RA
IL12A

IL13
IL13RA2
IL17A
IL17RA
IL18
IL19
IL1A
IL1B
IL1B
IL1R1
IL1RAP
IL2
IL20RA
IL33
IL4
IL4R
IL6
IL6R
IL7
ILK
INCA1
INF2
ING2
ING5
INHBA
INHBB
INS
INSRR
IQGAP1
IREB2
IRF1
IRF2
IRF9
IRGM
ITGA2
ITGA3
ITGA4
ITGA5
ITGA6
ITGA8
ITGAM
ITGAV
ITGB1
ITGB3
ITGB4
ITM2C
ITPR1
ITPR3
ITPRIP
IVNS1ABP
JAK1

JAK2
JAK3
JMY
JNK
JUN
KAT5
KAT8
KDM1A
KDM3A
KDM4A
KDR
KEAP1
KIF1B
KIF25
KIF2C
KIT
KITLG
KL
KLF12
KLHL22
KLHL3
KRAS
KRT18
KRT8
KXD1
LACRT
LAMA3
LAMB3
LAMC2
LAMP1
LAMP2
LAMP3
LAMTOR1
LAMTOR2
LAMTOR3
LAMTOR4
LAMTOR5
LAPTM4A
LAPTM4B
LAPTM5
LARP1
LAT
LAT2
LATS1
LCK
LEP
LEPR
LGALS1
LGALS12
LGALS3
LGALS8

LGALS9
LGMN
LIAS
LIPA
LIPT1
LIPT2
LMO3
LPAR1
LPAR2
LPCAT3
LRP1
LRPPRC
LRRK2
LRSAM1
LTB4R2
LTBR
LTF
LY96
LYN
LZTS1
M6PR
MADD
MAEL
MAGEA3
MAN2B1
MANBA
MAP1LC3
A
MAP1LC3
B
MAP1LC3
C
MAP2K1
MAP2K2
MAP2K5
MAP3K5
MAP3K7
MAP6
MAPK1
MAPK11
MAPK14
MAPK15
MAPK3
MAPK7
MAPK8
MAPK8IP1
MAPK8IP2
MAPK9
MAPT
MARCHF7
MARK4
MAVS

MAZ
MCL1
MCOLN1
MCU
MDM2
MEFV
MEGF11
MELK
MET
MFF
MFN2
MFSD8
MGAT5
MIA
MID2
MIF
MILR1
MIR10A
MIR124-1
MIR132
MIR141
MIR145
MIR15A
MIR16-1
MIR17
MIR1827
MIR198
MIR199A1
MIR200A
MIR200B
MIR200C
MIR204
MIR21
MIR210
MIR221
MIR222
MIR26A1
MIR26B
MIR27B
MIR30B
MIR30C1
MIR363
MIR449A
MIR525
MIR6744
MIRLET7B
MKNK2
MLH1
MLKL
MLLT11
MLST8

MMP1
MMP11
MMP13
MMP2
MMP9
MNT
MNX1
MOAP1
MPO
MPV17L
MRGPRX2
MRTFA
MSH2
MSH6
MSLN
MSX1
MT1G
MT3
MTA1
MTCL1
MTDH
MTF1
MTM1
MTMR3
MTMR4
MTMR8
MTMR9
MTOR
MTUS2
MUC1
MUL1
MYBBP1A
MYC
MYD88
MYH10
MYH14
MYH9
MYL6
MYLK
MYO5A
NACC2
NAGA
NAGLU
NAGPA
NANOS3
NAPSA
NAT1
NBN
NCK1
NCK2
NCKAP1

NCOA4
NCX1
NDEL1
NDUFA11
NDUFA13
NDUFS1
NDUFS3
NEDD4
NEU1
NFATC4
NFE2L2
NFKB1
NFKB1A
NFS1
NGF
NGFR
NKX3-1
NLE1
NLRC4
NLRP1
NLRP2
NLRP3
NLRP6
NLRP7
NME5
NMT1
NOC2L
NOD1
NOD2
NOG
NOL3
NONO
NOS3
NOTCH1
NOTCH3
NOX1
NOX2
NOX3
NOX4
NPC1
NPC2
NPRL2
NQO1
NR4A2
NR4A3
NRAS
NRBP2
NRF2
NT5C
NT5E
NTF3

NTRK1
NTRK2
NUBPL
NUPR1
OGG1
OLFM3
OPA1
OPTN
ORMDL3
OSBPL7
OTUB1
OXSM
P2RX4
P2RX7
P4HB
PAAN
PAD4
PADI4
PAFAH1B2
PAK1
PAK2
PAK3
PAK4
PAK5
PARK7
PARP
PARP1
PARP2
PAWR
PBK
PCBP1
PCBP2
PCGF2
PCK2
PDCD10
PDCD4
PDCD5
PDCD6
PDCD6IP
PDGFB
PDGFRB
PDHA1
PDHB
PDIA3
PDK1
PDK2
PDK4
PDLIM1
PDPK1
PDX1
PEA15

PEBP1
PELI3
PERP
PF4
PFKFB3
PGAM5
PGD
PHB
PHB2
PHF23
PHIP
PHKG2
PHLDA2
PHLDA3
PI3KC3
PI4KB
PIAS4
PIDD1
PIH1D1
PIK3C2A
PIK3C3
PIK3CA
PIK3CB
PIK3CD
PIK3CG
PIK3R1
PIK3R2
PIK3R3
PIKFYVE
PIM2
PIN1
PINK1
PIP4K2A
PIP4K2B
PIP4K2C
PIP4P1
PJVK
PKCB
PLA2G15
PLA2G3
PLA2G4
PLA2G7
PLAGL2
PLAT
PLAU
PLAUR
PLCG1
PLEKHF1
PLEKHM1
PLEKHM2
PLG

PLK1
PLK2
PLK3
PLPP2
PLSCR3
PMAIP1
PML
POLB
POLDIP2
POU4F1
POU4F2
PPARD
PPARG
PPIA
PPID
PPIF
PPM1F
PPP1CA
PPP1R13B
PPP1R15A
PPP2CA
PPP2R1A
PPP2R1B
PPP2R2A
PPP2R2D
PPP2R5A
PPP3CA
PPP3CC
PPP3R1
PPT1
PPT2
PRAG1
PRDX2
PRDX4
PRELID1
PRF1
PRKAA1
PRKAA2
PRKAB1
PRKAB2
PRKACA
PRKACG
PRKAG1
PRKAG2
PRKAG3
PRKCA
PRKCD
PRKCI
PRKCQ
PRKCZ
PRKD1

PRKDC
PRKN
PRKRA
PRNP
PRODH
PROM2
PRPF4B
PRTN3
PSAP
PSAPL1
PSEN1
PSMD10
PSME3
PTEN
PTGDR
PTGDS
PTGIS
PTGS2
PTH
PTHLH
PTK2
PTK2B
PTK6
PTPMT1
PTPN1
PTPN11
PTPN2
PTPN22
PTPRC
PTRH2
PTTG1IP
PYCARD
PYG
QARS1
QSOX1
RAB34
RAB39B
RAB3A
RAB3GAP
1
RAB3GAP
2
RAB7A
RAB8A
RAC1
RAC2
RACK1
RAD9A
RAF1
RALB
RANBP9
RASIP1

RB1
RB1CC1
RBCK1
RBL2
RELA
RET
RETREG1
RETREG3
RFFL
RGR
RHEB
RHOA
RHOB
RHOG
RHOT1
RHOT2
RIPK1
RIPK2
RIPK3
RMC1
RNF146
RNF152
RNF181
RNF183
RNF186
RNF31
RNF34
RNF41
RNF5
ROCK
ROCK1
RPL11
RPL26
RPL8
RPN1
RPS27L
RPS3
RPS6KB1
RPS7
RPTOR
RRAGA
RRAGB
RRAGC
RRAGD
RRP8
RTKN2
RTL10
RUBCN
RUBCNL
RUFY4
RYR1

RYR2
S100A13
S100A4
S100A8
S100A9
SAT1
SAT2
SATB1
SCAF11
SCARB2
SCFD1
SCG2
SCN2A
SCOC
SCRT2
SDCBP
SEC22B
SELENOK
SELENOS
SEMA7A
SENP1
SEPTIN4
SERINC3
SERPINA1
SERPINE1
SESN1
SESN2
SESN3
SFN
SFPQ
SFRP1
SFRP2
SGMS1
SGPL1
SGPP1
SGSH
SH3BP4
SH3GLB1
SH3RF1
SHARPIN
SHC1
SHH
SHISA5
SIAH1
SIAH2
SIK1
SIK2
SIRPA
SIRT1
SIRT2
SIRT3

SIVA1
SKIL
SKP2
SLC11A1
SLC11A2
SLC17A5
SLC1A5
SLC25A4S
SLC25A5
SLC2A1
SLC31A1
SLC35F6
SLC38A9
SLC39A14
SLC39A8
SLC3A2
SLC40A1
SLC7A11
SLC9A3R1
SMAD3
SMAD4
SMCR8
SMG1
SMPD1
SNAI1
SNAI2
SNAP23
SNAPIN
SNCA
SNRNP70
SNW1
SNX16
SNX32
SNX4
SNX5
SNX6
SOD1
SOD2
SOGA1
SOGA3
SORL1
SORT1
SP1
SP100
SPAG9
SPATA2
SPHK2
SPIB
SPINK1
SPTLC1
SPTLC2

SQLE
SQSTM1
SRC
SREBF1
SREBF2
SRPX
SST
SSTR3
SSTR5
ST20
STAT1
STAT2
STAT3
STAT4
STAT5A
STAT5B
STAT6
STBD1
STEAP3
STING1
STK11
STK24
STK25
STK3
STK4
STRADB
STUB1
STX4
STXBP1
STXBP2
STYXL1
SUMF1
SUPT5H
SVIP
SYK
SYNPO2
SYTL4
SYVN1
TAAR5
TAAR9
TAB2
TAB3
TAF9
TAF9B
TBC1D14
TBC1D25
TBK1
TCF7L2
TCIRG1
TERT
TEX264

TF
TFDP1
TFDP2
TFEB
TFPT
TFRC
TGFB1
TGFB2
TGFBR1
THBS1
TICAM1
TICAM2
TIGAR
TIMM50
TIMP1
TIMP3
TINAP
TIRAP
TIRP
TLE1
TLE5
TLK2
TLN1
TLR2
TLR3
TLR4
TM2D1
TMBIM1
TMBIM6
TMC8
TMEM102
TMEM106
B
TMEM109
TMEM117
TMEM14A
TMEM150
A
TMEM150
B
TMEM150
C
TMEM161
A
TMEM39A
TMEM39B
TMEM59
TNF
TNFAIP3
TNFRSF10
A
TNFRSF10
B

TNFRSF10
C
TNFRSF12
A
TNFRSF19
TNFRSF1A
TNFRSF1B
TNFRSF25
TNFRSF6
TNFSF10
TNFSF12
TNFSF6
TNK2
TOMM7
TOPORS
TP53
TP53BP2
TP53INP1
TP53INP2
TP63
TP73
TPCN1
TPCN2
TPD52L1
TPM1
TPP1
TPT1
TRADD
TRAF1
TRAF2
TRAF5
TRAF7
TRAP1
TREM2
TRIAP1
TRIB3
TRIF
TRIM13
TRIM14
TRIM21
TRIM22
TRIM27
TRIM31
TRIM32
TRIM34
TRIM38
TRIM39
TRIM5
TRIM6
TRIM65
TRIM68
TRIM8

TRIML1
TRIML2
TRPM7
TSC1
TSC2
TSG101
TSPO
TUBB3
TWIST1
TXNDC12
TYK2
TYROBP
UACA
UBA5
UBB
UBE2C
UBE2K
UBE2U
UBE4B
UBQLN1
UBQLN2
UBQLN4
UCHL1
UFC1
UFL1
UFM1
ULK1
UMOD
UNC13D
UNC5B
UQCRC1
URI1
USP10
USP13
USP21
USP28
USP30
USP33
USP36
USP47
UVRAG
VAMP7
VAMP8
VDAC1
VDAC2
VDAC3
VEGFA
VNN1
VPS13C
VPS13D
VPS24

VPS26A
VPS26B
VPS33A
VPS33B
VPS35
VPS37A
VPS4
VPS4A
VTN
WAC
WASH3P
WASHC1
WDFY3
WDR24
WDR35
WDR41
WDR6
WDR81
WIPI2
WNT4
WWOX
XBP1
XIAP
YAP1
YBX3
YWHAB
YWHAE
YWHAG
YWHAH
YWHAQ
YWHAZ
ZBP1
ZBTB7A
ZC3H12A
ZC3HC1
ZDHHC3
ZEB1
ZEB2
ZFYVE16
ZKSCAN3
ZMPSTE24
ZMYND11
ZNF205
ZNF304
ZNF32
ZNF385A
ZNF385B
ZNF622
ZSWIM2
